# Integrative pan-cancer analysis reveals AARS2 as a lactylation-associated biomarker and therapeutic target in colon adenocarcinoma

**DOI:** 10.3389/fimmu.2026.1732811

**Published:** 2026-02-27

**Authors:** Mingyang Zou, Zixuan Ding, Yifan Fu, Wenxin Yu, Yulan Song, Xinyue Wu, Yixin Pan, Shaobo Wu, Jiebin Pan

**Affiliations:** 1 The Second Hospital & Clinical Medical School, Lanzhou University, Lanzhou, Gansu, China; 2 The First Clinical Medical College, Lanzhou University, Lanzhou, China; 3 Department of Oncology, Tongji Hospital Affiliated to Tongji Medical College of Huazhong University of Science and Technology, Wuhan, Hubei, China; 4 Department of Pathology, The Second Hospital & Clinical Medical School, Lanzhou University, Lanzhou, Gansu, China; 5 Department of Orthopaedics, The Second Hospital & Clinical Medical School, Lanzhou University, Lanzhou, Gansu, China; 6 Department of General Surgery, The Second Hospital & Clinical Medical School, Lanzhou University, Lanzhou, Gansu, China

**Keywords:** AARS2 gene, colon adenocarcinoma (COAD), lactylation (Kla), Pan-cancer analysis, prognostic biomarker, Tumor microenvironment - TME

## Abstract

**Background:**

Colon adenocarcinoma (COAD) is a lethal malignancy with a poor prognosis. The tumor microenvironment (TME) is pivotal in its development, within which lactate accumulation is a common metabolic hallmark. Lactylation, a novel post-translational modification driven by lactate, serves as a crucial link between tumor metabolism and immunosuppression. It plays multifaceted roles in promoting malignant progression, immune evasion, and chemoresistance. Therefore, systematically investigating lactylation and identifying its key mediators may yield novel therapeutic targets and strategies for COAD.

**Methods:**

We performed an integrative multi-omics analysis of lactylation-related genes (LRGs) in COAD and pan-cancer cohorts, leveraging bulk RNA-seq, single-cell RNA-seq, and spatial transcriptomic data from public repositories including TCGA and GEO. A curated set of 160 LRGs was investigated using a multi-step machine learning framework, integrating Cox regression, time-dependent ROC analysis, and optimal risk stratification to construct robust prognostic signatures, which collectively identified AARS2 as a pivotal candidate. Subsequent multi-faceted oncogenic characterization of AARS2 encompassed its expression profiles, diagnostic and prognostic value, and associations with tumor microenvironment heterogeneity. To validate AARS2 at the protein level, we first interrogated the HPA database and subsequently confirmed its expression using immunohistochemistry (IHC) on an independent cohort of clinical COAD specimens. To further elucidate AARS2’s functional role in COAD pathogenesis and its potential linkage to lactylation biology, *in vitro* validation was performed using the human COAD cell line HCT116. AARS2 expression was confirmed at both protein (Western blot) and mRNA (qRT-PCR) levels. qRT-PCR additionally quantified transcriptional changes in immune-related genes (CCL5, CXCL10, IFNB1), while extracellular lactate accumulation was measured to assess AARS2-associated metabolic alterations. All statistical analyses were performed in R, with a significance threshold of p < 0.05.

**Results:**

Integrative multi-omics analyses identified AARS2 as significantly upregulated in COAD and multiple malignancies, with elevated expression correlating with adverse clinical outcomes. Single-cell and spatial transcriptomic profiling indicated predominant enrichment of AARS2 in malignant cell populations and association with immunosuppressive microenvironment features. Functional enrichment suggested potential involvement in epithelial–mesenchymal transition, hypoxia response, and cell cycle pathways. Immunohistochemical validation in clinical COAD specimens confirmed higher AARS2 protein levels in tumor tissues versus adjacent normal mucosa; concurrent elevation of cGAS protein was observed, though functional activity requires contextual interpretation. *In vitro* studies in HCT116 cells revealed that AARS2 knockdown reduced extracellular lactate accumulation and attenuated global protein lactylation. Concomitantly, transcriptional upregulation of cGAS–STING pathway-associated genes (CCL5, CXCL10, IFNB1) was observed following AARS2 silencing. These correlative findings suggest a potential nexus between AARS2, lactate metabolism, protein lactylation dynamics, and innate immune signaling modulation in COAD.

**Conclusion:**

AARS2 correlates with lactylation dynamics, metabolic features, and immune modulation in COAD, suggesting a potential role in lactylation-associated cGAS–STING pathway regulation. These correlative observations warrant rigorous mechanistic validation to define causality and assess translational potential.

## 1 Introduction

Colon adenocarcinoma (COAD) is a common malignant tumor arising from the glandular epithelial cells of the colon, and is one of the leading causes of cancer-related deaths worldwide (1). Its etiology involves multifactorial interactions with a complex pathogenesis. Risk factors include age, family history, inflammatory bowel disease, lifestyle factors such as poor dietary habits (2), specific bacterial infections and gut dysbiosis (3, 4). Meanwhile, the tumor microenvironment plays a crucial role in the occurrence and development of colorectal cancer (5–7). Among them, the Warburg effect, as a significant metabolic characteristic of tumors, enables cancer cells to preferentially undergo glycolysis under oxygen-rich conditions, generating a large amount of lactic acid (6, 8). This process not only directly promotes immunosuppression and drug resistance through complex feedback mechanisms, but also drives tumor progression by activating the glycolysis pathway, influencing genetic mutations and reshaping the metabolic state of the microenvironment (8–10). This metabolic reprogramming further enhances tumor immune evasion, allowing cancer cells to interact with immune cells in the tumor microenvironment, ultimately forming a highly suppressed tumor immune microenvironment (11). Thus, lactic acid is the core mediator connecting tumor metabolism and immunosuppression (12). The realization of this effect largely depends on protein lactylation modification (13).

Lactylation is a novel post-translational modification (PTM) driven by the metabolite lactate (14). Its discovery has unveiled new biological functions of lactate that transcend its traditional identity as a metabolic waste product, expanding our understanding of its roles beyond metabolic regulation (15). Mediated by lactate as a metabolic intermediate, this modification occurs on lysine residues of both histone and non-histone proteins (16). It is reversibly regulated by writer and eraser enzymes, thereby influencing gene transcription, protein function, and the epigenetic landscape (15, 17). In cancer biology, lactylation plays a pivotal role as a central component of tumor metabolic reprogramming (the Warburg effect), enabling cancer cells to adapt to nutrient-constrained microenvironments (18). Given that its reversibility is intricately linked to the metabolic state of tumor cells, lactylation has emerged as a potential pharmacological intervention target, offering novel therapeutic avenues for cancer treatment (18, 19). Notably, the stability of mitochondrial alanyl-tRNA synthetase (AARS2) is regulated by oxygen levels (20). Under hypoxic conditions, AARS2 has been shown to mediate lactylation of mitochondrial proteins, thereby suppressing oxidative phosphorylation (OXPHOS) and helping cells sustain metabolic homeostasis in low-oxygen environments (21). This mechanism directly establishes a functional link between lactylation and cellular energy adaptation (22, 23).

In COAD, lactylation plays a critical role (24, 25). Its global modification levels are significantly upregulated in tumor tissues and closely associated with malignant progression and poor prognosis, serving as an independent prognostic biomarker for the disease (26, 27). At the molecular level, lactylation drives tumor invasion and metastasis by activating the PI3K/AKT signaling pathway to promote epithelial-mesenchymal transition (EMT) in colon cancer cells (28). It is particularly notable that in colon cancer stem cells (CCSCs), histone lactylation has been demonstrated to significantly enhance chemoresistance—a phenomenon validated in both *in vitro* cellular models and *in vivo* animal models (29–31). Furthermore, lactylation profoundly modulates the tumor immune microenvironment (TIME) by promoting immune escape and influencing immunotherapy response—via pathways such as facilitating macrophage polarization toward the M2 phenotype and regulating lactate levels to suppress Migration Inhibitory Factor (MIF) (26, 28). These studies underscore the multifaceted impact of lactylation in COAD, providing a theoretical basis for developing targeted combination therapies, such as synergized with chemotherapy (32).

However, the roles of the lactylation and the AARS2 gene in the progression of COAD remain poorly understood, which hinders the development of targeted therapeutic strategies. This study aims to elucidate the diagnostic and prognostic value of AARS2 in COAD by integrating bioinformatics analyses with experimental validation *in vitro*.

## 2 Methods

### 2.1 Data acquisition and preparation

Pan-cancer and COAD-specific analyses were performed using systematically curated public omics datasets. RNA sequencing (RNA-seq) profiles and annotated clinical metadata were acquired from TCGA database (https://portal.gdc.cancer.gov/) and GEO repositories (https://www.ncbi.nlm.nih.gov/geo/). Pan-cancer cohorts included multiple datasets, including GSE103479/GSE104645/TCGA-GTEx/GSE71187/GSE9893/GSE107943/GSE63624/GSE10846/GSE181063/GSE54236/GSE28735/GSE17679/GSE190113/GSE98394/GSE99898/GSE28541/GSE84426 (33). Non-neoplastic tissues and samples with censored survival outcomes (overall survival < 30 days or missing clinical data > 20%) were excluded. All gene annotations were standardized to gene symbols to ensure analytical consistency.

Single-cell RNA sequencing (scRNA-seq) data were employed to characterize tumor microenvironment heterogeneity and cellular ecosystem dynamics. Two datasets (EMTAB8107 and GSE166555) were analyzed using standardized preprocessing workflows (cells retained if 200 < nFeature_RNA < 6,000 and mitochondrial gene percentage < 20%), which included quality control, dimensionality reduction, cluster annotation, and identification of malignant cells. All analytical procedures adhered to TISCH2 standards to ensure reproducibility (34).

Spatially resolved transcriptomic profiles were acquired using the 10X Visium Spatial Transcriptomics platform (10x Genomics, available at: https://www.10xgenomics.com/resources/datasets). The data were derived from FFPE-preserved colorectal carcinoma sections (samples CRC1, CRC2, and CRC3), facilitating the integrated analysis of histopathological structure and molecular signatures within the tumor microenvironment.

All data analyzed in this study were obtained from publicly available sources, including previously published studies and established databases.

### 2.2 Machine learning model construction and gene selection

A curated set of 160 lactylation-related genes (LRGs) was compiled through systematic integration of GSEA Molecular Signatures Database, KEGG pathway annotations, and manual curation of recent literature (**Supplementary Dataset 1**). Within the COAD cohort, univariate Cox proportional hazards regression analysis was initially performed on all 160 genes without multiple testing correction as an exploratory screening step to generate a broad candidate pool for subsequent rigorous validation. This approach was deliberately adopted to preserve biologically plausible candidates that might be excluded under stringent correction at the screening stage. Core prognostic candidates were subsequently refined through a multi-algorithm consensus framework specifically applied to the COAD cohort. Five independent machine learning approaches—Lasso regression, Ridge regression, Elastic net, Stepwise Cox regression, and CoxBoost—were applied to identify genes with consistent prognostic significance. Risk scores were calculated as the linear combination of gene expression values weighted by algorithm-derived coefficients. Model performance was evaluated using time-dependent receiver operating characteristic (ROC) analysis at 1-, 3-, and 5-year survival time points. An optimal risk stratification cutoff was determined using maximally selected rank statistics (via the survminer R package), with statistical significance of survival differences assessed by log-rank testing. Differential expression patterns of selected genes between risk groups were visualized using heatmaps (pheatmap R package). All analyses were performed in R with packages survival, glmnet, CoxBoost, and timeROC.

### 2.3 Gene expression analysis

Expression matrices were processed using tumor-type-specific normalization pipelines. For pan-cancer analyses, z-scores were calculated based on cohort-specific means and standard deviations. Differential expression between tumor and normal samples was assessed using non-parametric tests with false discovery rate control (limma v3.56.2; |log_2_FC| > 1, P < 0.05).

Diagnostic accuracy was evaluated via receiver operating characteristic (ROC) analysis. Area under the curve (AUC) with 95% confidence intervals and smoothed ROC curves were generated to determine discriminatory power.

### 2.4 Survival prognosis analysis

Prognostic associations were assessed using Cox proportional hazards models, with verification of proportional hazards assumptions. Univariate analysis provided hazard ratios (HR) and 95% confidence intervals (95% CI). External validation was conducted on independent GEO cohorts using the same modeling framework.

For the COAD meta-analysis, a fixed-effects model was employed to combine HR, with inverse variance weighting. Genes significantly associated with improved survival (HR < 1) were considered potential tumor suppressors, while those associated with poorer survival (HR > 1) were considered potential oncogenes. Multivariable Cox models incorporating clinical covariates were developed and visualized using forest plots.

### 2.5 Tumor microenvironment analysis

The immune microenvironment was characterized using three complementary approaches: single-sample Gene Set Enrichment Analysis (ssGSEA; GSVA v1.48.2, kcdf=“Gaussian”), xCell deconvolution, and CIBERSORTx (LM22 signature, 1,000 permutations) (35, 36). Patients were stratified into expression-based quartiles for further analysis. Infiltration scores were filtered and subsequently clustered using unsupervised hierarchical clustering, which revealed conserved immune subtypes across malignancies.

### 2.6 Enrichment analysis

Functional annotation was carried out using integrated Gene Ontology (GO), Kyoto Encyclopedia of Genes and Genomes (KEGG), and Gene Set Enrichment Analysis (GSEA; clusterProfiler v4.8.2, Org.Hs.eg.db v3.17.0; 1,000 permutations, gene set size 15–500, q < 0.05) (37–39). The TCGA-COAD cohort was divided into high- and low-expression groups based on median expression of target genes for subsequent functional investigations. Differential gene expression analysis was performed using the “limma” package, with pre-defined thresholds for fold-change and statistical significance (40).

Functional states of malignant cells were further characterized using the CancerSEA database to explore various oncogenic phenotypes (41). And, pathway activity was quantified using z-score-based algorithms, and functional state scores were computed via the “GSVA” package (42). Normalized scores were standardized and subjected to correlation analysis to evaluate associations between gene expression and functional states.

### 2.7 Single-cell RNA-seq analysis

Single-cell transcriptomic profiling was performed using the Seurat package following standard analytical workflows (43). Cells were subjected to quality control based on thresholds for unique molecular identifiers and mitochondrial gene content. Preprocessing included normalization, dimensionality reduction via principal component analysis, and batch effect correction. Cells were stratified into high- and low-expression groups based on median expression levels, and cluster proportions were compared across conditions (44).

Cell-cell communication analysis was performed to infer ligand-receptor interaction networks. Interaction probabilities and communication strengths were quantified between cellular subpopulations. Directional signaling flux and core pathways were identified based on signal input/output patterns and centrality metrics (45).

### 2.8 Spatial transcriptomic analysis

Spatially resolved transcriptomic profiling was employed to investigate the topographic distribution and functional relevance of target genes within the tumor microenvironment (46). Cell type deconvolution of spatial transcriptomics data was performed using a reference-based Bayesian framework. Quality control was conducted according to standard scRNA-seq processing criteria.

A gene signature score matrix was generated by averaging expression of top marker genes from single-cell references. Cellular abundance gradients across spatial domains were quantitatively assessed and visualized using color gradients to represent enrichment intensity. Histological regions were classified as malignant based on predefined cellular composition thresholds, facilitating patient stratification into normal-like and mixed subgroups. Differential expression between groups was evaluated using non-parametric tests with multiple testing correction.

### 2.9 Protein-protein interaction and functional analysis

The Human Protein Atlas database (https://www.proteinatlas.org) provides comprehensive mRNA and protein expression data across 44 human tissues, along with antibody-based protein profiles that include expression levels and subcellular localization information (47).

To identify and predict interactions among genes or proteins, we utilized the STRING database (http://string-db.org/). Additionally, BioGRID version 4.4 (https://thebiogrid.org/) was employed as a key resource for accessing high-quality, curated, and non-redundant physical and genetic protein-protein interactions (PPIs).

A protein-protein interaction network was constructed using a combined score cutoff of > 0.9. Based on this network, the expression of AARS2-related proteins was further analyzed across multiple cancer types.

### 2.10 Immunohistochemistry analysis

Immunohistochemical analysis was performed to assess AARS2 protein expression in COAD tissue specimens. The study protocol received approval from the Ethics Committee of the Second Hospital of Lanzhou University (Approval No. 2025A-1084). Tissue samples were collected from 12 patients diagnosed with COAD, with adjacent normal colon mucosa serving as controls. All specimens were promptly snap-frozen in liquid nitrogen and transferred to the laboratory for subsequent analysis. Comprehensive clinical metadata for the cohort—including gender/age distribution, TNM staging, histological grade, prior treatment history, and MSI status (MSI-H versus MSS)—along with correlation analyses evaluating associations between AARS2 protein expression levels and key clinicopathological parameters (T stage, N stage, M stage, and tumor grade), are fully documented in the **Supplementary Materials** (**Supplementary Table 1**).

For IHC, a primary antibodys AARS2 (Proteintech, 22696-1-AP) and cGAS (Proteintech, 26416-1-AP) were applied at a dilution of 1:50. Protein expression was quantitatively evaluated using the ImageJ IHC profiler plugin. The assessment integrated both staining intensity and the proportion of immunopositive areas. The percentage of positive staining relative to the total tissue area (distribution) was calculated, and a final expression score was derived by multiplying the intensity value by the distribution percentage.

Based on the median expression score across all samples, specimens were classified into high- and low-expression groups: scores above the median were defined as high expression, while those equal to or below the median were considered low expression. This classification was utilized in subsequent univariate and multivariate regression analyses. To ensure objectivity, three independent pathologists performed the evaluations blinded to all clinical data.

### 2.11 Cell culture

To investigate the functional role of AARS2 in COAD at the cellular level, human COAD cell lines (HCT 116) were used in this study. All cell lines were obtained from authenticated cell banks and routinely tested to exclude mycoplasma contamination.

Cells were cultured in the recommended complete growth medium (DMEM) supplemented with 10% fetal bovine serum and 1% penicillin–streptomycin, and maintained at 37°C in a humidified incubator with 5% CO_2_. Cells within 20 passages were used for all subsequent experiments to ensure reproducibility and stability of cellular phenotypes.

### 2.12 Western blot

Western blot analysis was performed to evaluate the protein expression levels of AARS2 in COAD cells, thereby supporting the transcriptomic findings at the protein level.

Cells were washed twice with ice-cold phosphate-buffered saline (PBS) and lysed in RIPA buffer supplemented with protease and phosphatase inhibitors. Cell lysates were clarified by centrifugation at 12,000 × g for 15 min at 4 °C. Protein concentrations were determined using a BCA protein assay kit. Equal amounts of protein (20–40 μg) were separated by SDS–PAGE and transferred onto PVDF membranes.

Membranes were blocked with 5% non-fat milk and incubated with primary antibodies overnight at 4 °C. After incubation with horseradish peroxidase-conjugated secondary antibodies, protein bands were visualized using enhanced chemiluminescence (ECL), with GAPDH and Tubulin used as an internal loading control.

### 2.13 Quantitative real-time PCR

Total RNA was extracted from HCT116 cells using TRIzol reagent. RNA concentration and purity were assessed spectrophotometrically (A260/A280 ratio 1.8–2.0). First-strand cDNA was synthesized from 1 μg total RNA using a commercial reverse transcription kit. Quantitative real-time PCR was performed in triplicate with SYBR Green chemistry on a real-time PCR system. The mRNA expression levels of AARS2, CCL5, CXCL10, and IFNB1 were quantified using gene-specific primers (sequences provided in **Supplementary Table 2**). Amplification conditions: 95°C for 30 s; 40 cycles of 95 °C for 10 s and 60°C for 30 s. GAPDH was used as the endogenous reference gene for normalization of target gene expression. Relative expression was calculated using the 2^−ΔΔCt method. Amplification specificity was confirmed by melting curve analysis. All procedures adhered to MIQE guidelines.

### 2.14 Lactate assay

Extracellular lactate levels in cell culture supernatants were measured to evaluate lactate accumulation in COAD cells associated with AARS2 expression.

Cells were seeded in 6-well plates and maintained under standard culture conditions. Culture supernatants were harvested at 24 h and 48 h post-seeding, centrifuged (e.g., 300 ×g, 5 min, 4°C) to remove cellular debris, and lactate concentrations were quantified using a L-Lactic Acid (LA) Colorimetric Assay Kit (Elabscience, catalogue no.: E-BC-K130-M) following the manufacturer’s protocol. Lactate levels were calculated from a standard curve and normalized to total protein content (determined by BCA assay) or cell number.

### 2.15 Statistical analysis

Statistical analyses were conducted using R software (version 4.3.1) with appropriate packages. Differences in gene expression among spatially defined regions (tumor, normal, and mixed) were assessed using Wilcoxon rank-sum tests. For single-cell transcriptomic data, the Kruskal–Wallis H test was used to compare gene expression across cell types, and Spearman’s correlation was applied to evaluate associations between immune cell proportions and gene expression levels. Survival analysis was performed using Kaplan–Meier curves with log-rank tests. Univariate and multivariate Cox proportional hazards models were used to identify independent prognostic factors, with HR and 95% confidence intervals reported. A p-value < 0.05 was considered statistically significant.

## 3 Result

### 3.1 Validation and pan-cancer analysis of a LRGs multi-gene prognostic signature

**Figure 1:**
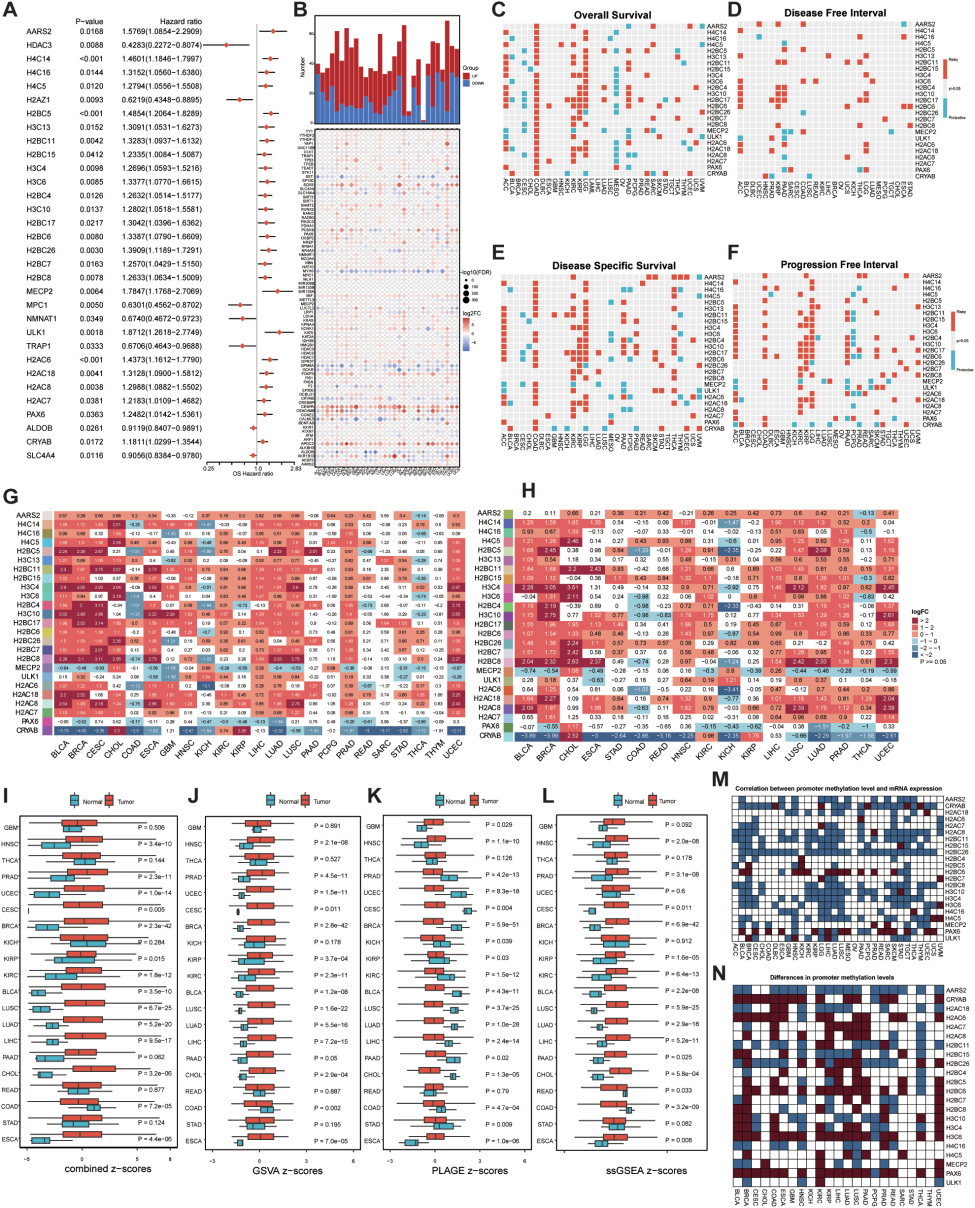
Expression differences and survival prognosis analysis of LRGs in pan-cancer and the characteristics of Lactylation. **(A)** 25 candidate genes are risk factors associated with an increased risk of death in COAD overall survival. **(B)** Multigenic differential analysis of TCGA-GTEx in pan-cancer. **(C)** Assessment of overall survival across pan-cancer using a polygenic univariate Cox model. **(D)** Assessment of disease free interval across pan-cancer using a polygenic univariate Cox model. **(E)** Assessment of disease specific survival across pan-cancer using a polygenic univariate Cox model. **(F)** Assessment of progression free interval AARS across pan-cancer using a polygenic univariate Cox model. **(G)** Pan-cancer differential expression analysis of a multi-gene signature. **(H)** Pan-cancer analysis of differentially expressed gene pairs. **(I)** Pan-cancer multi-gene enrichment analysis using Z-score. **(J)** Pan-cancer multi-gene enrichment analysis using GSVA. **(K)**Pan-cancer multi-gene enrichment analysis using ssGSEA. **(L)** Pan-cancer multi-gene enrichment analysis using PLAGE. **(M)** Correlation analysis of promoter methylation levels. **(N)** Differential analysis of promoter methylation levels.

Univariate Cox survival analysis was performed on the 160 lactylation-related genes across pan-cancer cohorts as an initial screening step to generate a candidate pool. Forest plots of hazard ratios (HR) and 95% confidence intervals identified 25 genes associated with overall survival in COAD (**Figure 1A**). Critically, these 25 genes represented an exploratory candidate pool rather than definitive prognostic markers. The complete statistical results for all 33 genes reaching nominal significance (p < 0.05) in the univariate Cox analysis—including gene ID, HR, HR.95L, HR.95H, and raw p-value—are provided in **Supplementary Table 3** for full transparency. Furthermore, we systematically characterized the RNA expression profiles of the aforementioned 160 genes across 30 distinct cancer types, leveraging a comprehensive integration of TCGA and GTEx datasets (**Figure 1B**). After successfully identifying 25 candidate genes associated with poorer OS in COAD, we further systematically evaluated the prognostic value of this gene set across a pan-cancer cohort encompassing 30 different cancer types. The analysis revealed that these candidate genes exhibit consistent prognostic patterns in several malignancies, including ACC, COAD, KIRC, KIRP, and LGG, where they were significantly associated with poorer outcomes in OS, DFI, DSS, and PFI, indicating a role as risk factors. In contrast, in tumors such as CESC and MESO, the same genes appeared to function as protective factors, correlating with more favorable clinical outcomes (**Figures 1C–F**). We next examined the expression patterns of these genes by comparing cancerous tissues with normal tissues across each cancer type, as well as by evaluating differences between tumor tissues and adjacent non-tumor tissues (**Figures 1G, H**). We also employed four distinct algorithms (Z-score, GSVA, ssGSEA) and PLAGE) to evaluate pathway activity associated with these core genes. The results indicated significantly altered pathway activity in a substantial number of tumor tissues compared to normal tissues (**Figures 1I–L**). Subsequently, we investigated the statistical correlation between promoter methylation levels and expression levels of these candidate genes associated with poorer OS in COAD across various cancer types, as well as compared their differences between tumor samples and normal controls (**Figures 1M, N**).

### 3.2 Identificated AARS2 as a key driver gene in COAD

**Figure 2:**
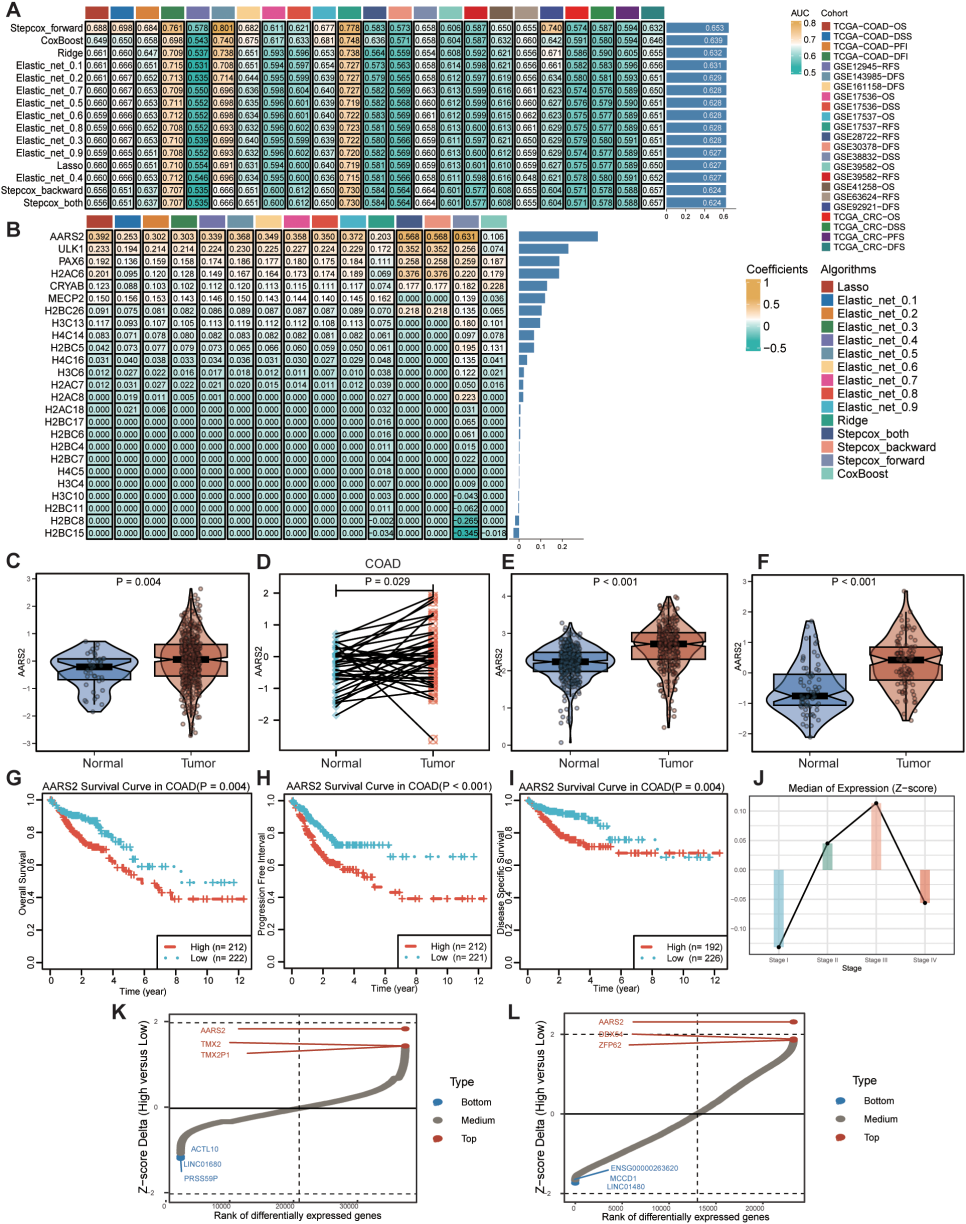
Prognostic value of the key lactylation-related gene AARS2 in COAD. **(A)** Mean AUC values at 1, 3, and 5 years for model evaluation using multiple algorithms. **(B)** Regression coefficient heatmap illustrating the contribution of 25 genes, with AARS2 identified as a key gene. **(C, D)** The AARS2 mRNA expression of paired COAD samples and unpaired COAD samples in TCGA-COAD cohort. **(E, F)** The validation of AARS2 mRNA expression of COAD samples in GSE71187 cohort and TCGA-GTEx cohort. **(G-I)** Survival differences between high and low AARS2 expression groups. Overall survival (OS), progression-free interval (PFI), and disease-specific survival (DSS). **(J)** Association between COAD Stage and AARS2 expression. **(K, L)** Identification of AARS2-associated upregulated genes in COAD datasets GSE103479 and GSE104645.

To develop robust prognostic models, we evaluated multiple algorithms—including regularized regression methods (Ridge, Lasso, Elastic_net with alpha values spanning 0.1–0.9), stepwise Cox models (Stepcox_forward, Stepcox_backward, Stepcox_both), and CoxBoost. We assessed their performance using the average area under the curve (AUC) at 1-, 3-, and 5-year time points across cancer types.Among these algorithms, the Stepcox_forward model demonstrated competitive performance: it showed relatively higher average AUC values in most cancer types and at most time points (**Figure 2A**). For example, the overall average AUC of Stepcox_forward across cancer types was 0.653—exceeding the average AUCs of other methods (e.g., CoxBoost: 0.639, Ridge: 0.632, Lasso: 0.627, and Elastic_net variants). Later, we utilized regression coefficient heatmaps to clarify the relative contributions of the 25 genes across distinct models (**Figure 2B**). Remarkably, AARS2 was identified as the gene with the largest regression coefficient across a wide range of algorithms, including Lasso, Ridge, Elastic_net with alpha values spanning from 0.1 to 0.9, stepwise Cox regression models (Stepcox_forward, Stepcox_backward, Stepcox_both), and CoxBoost. AARS2 was subsequently identified as the key gene for further analysis. Specifically, meta-analysis revealed that high AARS2 expression was significantly associated with poorer survival in COAD patients (pooled HR = 1.17, 95% CI: 1.03–1.33, p < 0.01; **Supplementary Figure 1A**).

To explore the potential role of AARS2 in carcinogenesis, we analyzed its mRNA expression in COAD using datasets from TCGA and GEO (GSE71187). Analysis of TCGA data indicated that AARS2 expression was significantly upregulated in COAD tissues relative to normal samples (P = 0.004; **Figure 2C**). This result was further supported by paired analysis, which also demonstrated elevated AARS2 expression in tumor tissues compared with matched adjacent non-tumor tissues (P = 0.029; **Figure 2D**). Consistent upregulation of AARS2 was also observed in independent validation cohorts from GEO and TCGA-GTEx (all P < 0.01; **Figures 2E, F**). These findings imply that AARS2 overexpression may contribute to the development of COAD.

Given the upregulated expression of AARS2, we further investigated its clinical relevance in COAD. Prognostic assessment and univariate Cox regression analyses were conducted using the TCGA cohort. Kaplan-Meier curves demonstrated that patients with high AARS2 expression exhibited significantly worse outcomes in overall survival (OS, P = 0.004), progression-free interval (PFI, P < 0.001), and disease-specific survival (DSS, P = 0.004) compared to those with low expression (**Figures 2G–I**). Consistent findings were observed across multiple independent validation datasets (**Supplementary Figures 1B–G**). Furthermore, univariate Cox regression confirmed AARS2 expression, T stage, and overall stage as independent prognostic factors for COAD (all P < 0.05; **Figure 2J**), underscoring the role of AARS2 as an independent predictor of patient survival. Subsequently, analysis of the GSE103479 and GSE104645 datasets revealed numerous differentially expressed genes, particularly those upregulated, which may be associated with AARS2-related functions and warrant further investigation (**Figures 2K, L**).

### 3.3 Expression of AARS2 in multiple tumor types

**Figure 3:**
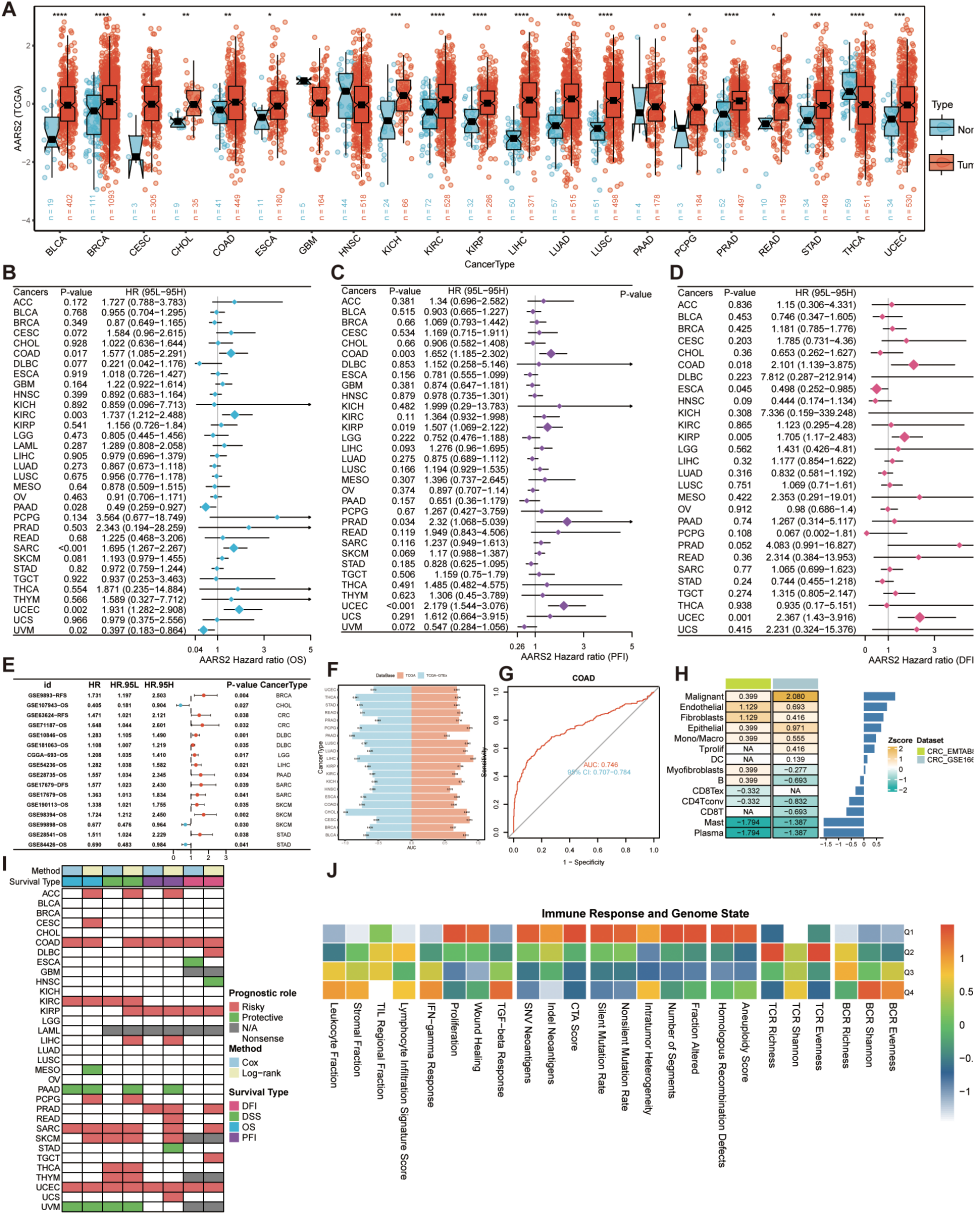
Pan-cancer analysis of AARS2. **(A)** Analysis of differential expression of AARS2 between tumor and normal tissues of multiple cancer types. **(B-D)** univariate Cox regression results for AARS2, including HR and confidence intervals for OS, DFl, and PFl. **(E)** validation of AARS2-associated survival outcomes across external datasets. **(F)** ROC curve analysis was used to evaluate the diagnostic accuracy of AARS2 in distinguishing tumors from normal tissues. **(G)** Diagnostic performance of AARS2 in COAD (AUC = 0.746). **(H)** Cellular heterogeneity of AARS2 expression in COAD, with predominant upregulation in malignant proliferating cells. **(I)** The context-dependent prognostic value of AARS2 across cancer types. **(J)** Heat map showing the average immune response and genomic status of cancers at different AARS2 expression levels. The symbols *, **, ***, and **** indicate statistical significance at the p < 0.05, p < 0.01, p < 0.001, and p < 0.0001 levels, respectively.

Through pan-cancer analysis of TCGA data, we observed significant differences in AARS2 expression between tumor and normal tissues across most cancer types (P < 0.05; **Figure 3A**). Overall, AARS2 expression showed substantial variation with a general trend toward overexpression in tumors. Specifically, AARS2 was significantly upregulated in BLCA, BRCA, CESC, CHOL, COAD, ESCA, KICH, KIRC, KIRP, LIHC, LUAD, LUSC, PCPG, PRAD, READ, STAD, and UCEC, while it was downregulated in THCA.

To assess the clinical relevance of AARS2, univariate Cox regression analysis was performed to estimate HR and 95% CI for overall survival (OS), disease-free interval (DFI), and progression-free interval (PFI) (**Figures 3B–D**). The results indicated that elevated AARS2 expression was associated with poorer survival outcomes across these endpoints. Moreover, as summarized in **Figure 3E**, AARS2 consistently functioned as a risk factor across multiple external datasets, further supporting that high AARS2 expression correlates with reduced survival. Together with ROC curve analysis demonstrating its accuracy in discriminating tumor from normal samples (**Figure 3F**), exemplified in COAD where AARS2 achieved an AUC of 0.746 (**Figure 3G**). These findings underscore the potential of AARS2 as both a prognostic and diagnostic biomarker. By integrating single-cell RNA sequencing data from the TISCH database (datasets EMTAB8107 and GSE166555), quantitative analysis demonstrated that the AARS2 gene displays pronounced cellular heterogeneity within COAD. This proliferation marker was specifically upregulated in the malignant proliferation subpopulation, with lower but detectable expression levels also observed in endothelial cells and fibroblasts (**Figure 3H**). Survival analysis summarized by heatmaps indicated that AARS2 serves as a risk factor in multiple external datasets, with patients exhibiting high AARS2 expression demonstrating poorer survival outcomes in ACC, COAD, KIRC, and KIRP. However, in certain tumor types, we observed the opposite trend, which may be attributable to their distinct biological contexts. For instance, elevated AARS2 expression was associated with a protective effect in PAAD and UVM (p < 0.001), while in several other cancers, no significant correlation with survival was detected. These findings underscore the context-dependent heterogeneity of AARS2’s prognostic impact across cancer types and suggest that further investigation is warranted to elucidate its precise mechanistic roles in different tumor environments (**Figures 3I, J**). Further links AARS2 expression to immunogenicity and DNA damage scores, providing more evidence for the role of AARS2 in immune regulation and genome stability.

Finally, single-cell and spatial transcriptomic analyses showed that AARS2 was predominantly expressed in malignant cells and was enriched in malignant microregions of tumor tissues (**Supplementary Figures 2A, B**). These findings highlight its potential role in shaping the tumor microenvironment and further highlight its importance in cancer biology.

### 3.4 Analysis of AARS2 overexpression in COAD based on spatial transcriptomics

Next, we explored its expression profile in CRC (colorectal cancer) from both transcriptomic and spatial transcriptomics perspectives.

**Figure 4:**
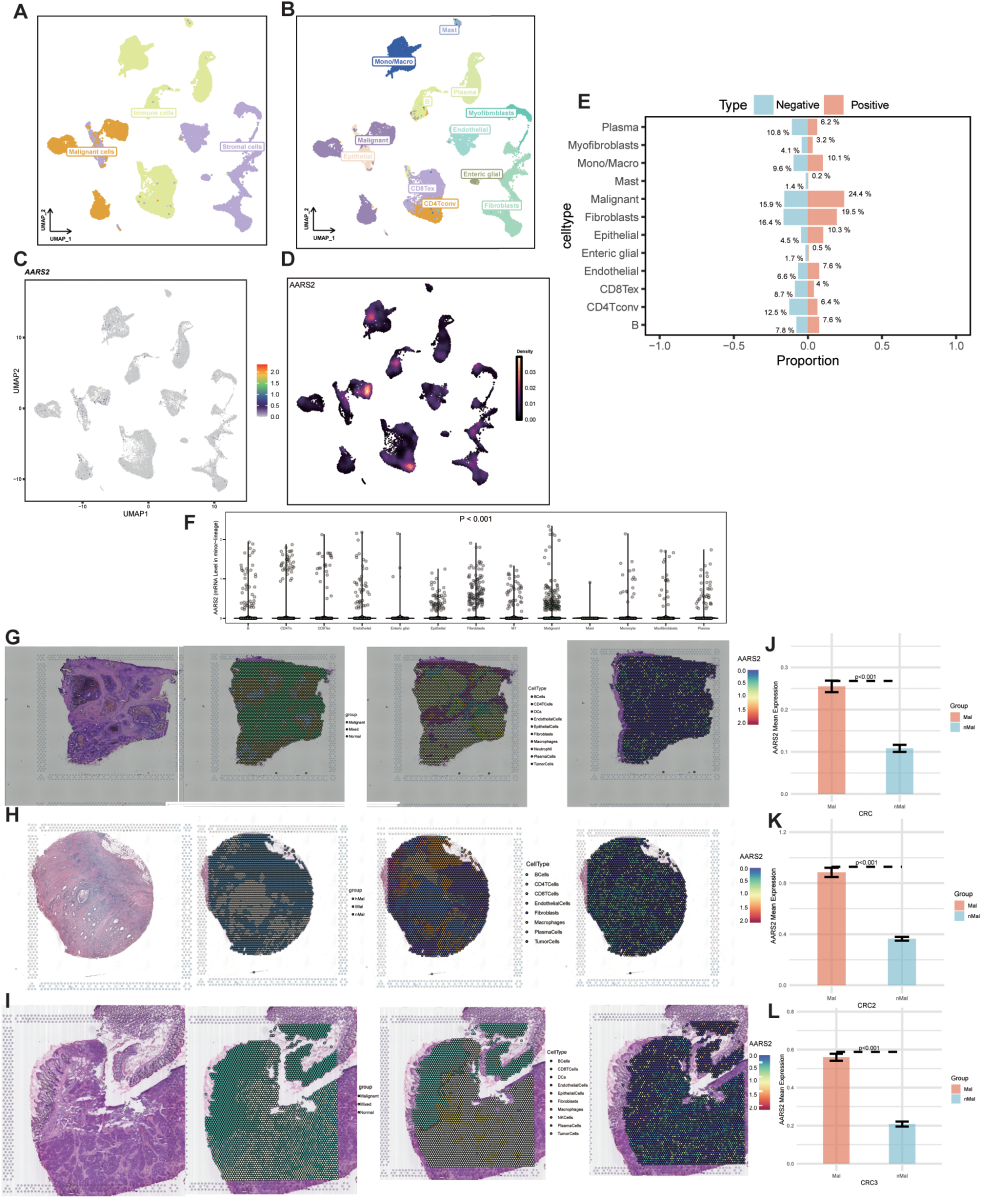
AARS2 enrichment in tumor cells promotes tumor progression. **(A, B)** UMAP visualization showing major and fine cellular lineages in the EMTAB8107 dataset. **(C, D)** Expression pattern of AARS2 across cell clusters. **(E)** Proportions of cell types in AARS2-positive and -negative populations. **(F)** AARS2 expression levels across diverse cell lineages. **(G-I)** Spatial co-localization of AARS2 expression with tumor regions in CRC samples. **(J-L)** Comparison of AARS2 expression between malignant and normal regions.

Following quality control, batch effect correction, and data normalization, the UMAP visualization clearly delineated cell clusters obtained from 21 COAD patients. The cells were classified into three major categories: malignant, stromal, and immune cells (**Figure 4A**). Subsequent subclustering identified distinct populations, including malignant cells, epithelial cells, fibroblasts, endothelial cells, B cells, exhausted CD8^+^ T cells (CD8Tex), helper T cells (Th), myofibroblasts, enteric glial cells, M1 and M2 macrophages, monocytes, mast cells, and plasma cells (**Figure 4B**). The UMAP visualization revealed prominent PAQR5 expression primarily within malignant tumor cells, macrophages, and monocytes (**Figures 4C, D**). We further quantified the proportions of each cell type in PAQR5-positive and AARS2-negative populations, observing a significantly higher percentage of malignant cells in the AARS2-positive group relative to the AARS2-negative group (**Figure 4E**). Moreover, differential expression analysis across cell types confirmed that AARS2 expression was largely restricted to malignant cells, fibroblasts, epithelial cells, and M1 macrophages (**Figure 4F**). This cell type-specific expression profile implies that AARS2 may promote COAD progression and influence macrophage immune functions. In the spatial transcriptomic analysis, we investigated the association between AARS2 and the TME. In all three samples (CRC1–3), AARS2 expression showed strong spatial co-localization with tumor cells, indicating its predominant presence within malignant regions (**Figures 4G–I**). By comparing predefined malignant and normal regions, we detected significantly elevated AARS2 expression in tumor areas, consistent with previous observations (**Figures 4J–L**). These findings suggest that AARS2 is closely linked to malignant cell populations in the TME and may contribute to colorectal cancer progression and local immune modulation.

### 3.5 AARS2 regulating immune landscape in the COAD

**Figure 5:**
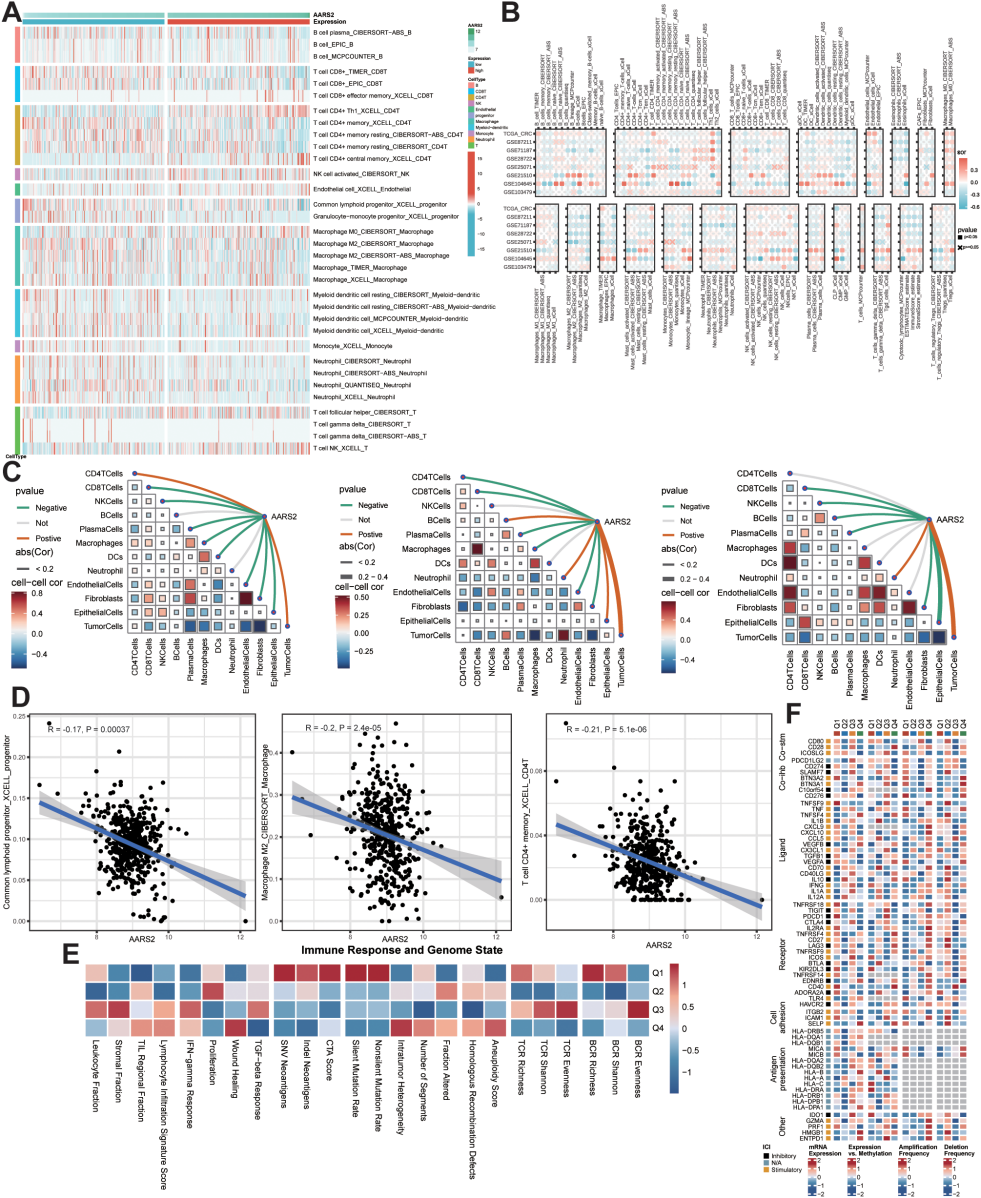
Analysis of the immune microenvironment of AARS2 in COAD. **(A)** Heatmap displaying differential immune cell infiltration between AARS2 high- and low-expression groups. **(B)** Spearman correlation analysis of AARS2 expression and immune cell infiltration levels. **(C)** Spearman correlation of AARS2 expression with tumor microenvironment components at spatial resolution. **(D)** Correlation of AARS2 with specific immune cell types assessed by XCELL and CIBERSORT algorithms. **(E)** Relationship between AARS2 expression and immune response or genomic status. **(F)** Landscape of immunomodulatory molecules associated with AARS2 expression.

We also used multiple algorithms to further explore the relationship between AARS2 expression and immune infiltration. The heatmap reveals distinct variations in immune cell composition across the sample groups, with markedly different distribution patterns between high- and low-expression cohorts (**Figure 5A**). Spearman correlation analysis revealed an inverse correlation between AARS2 and a wide range of immune-infiltrating cells in cancer, some of which were particularly strong (**Figure 5B**).

Spatial transcriptomic analysis was performed to assess the Spearman correlation between AARS2 expression and tumor microenvironment components at spatial resolution (**Figure 5C**). Consistent with prior localization findings, AARS2 expression correlated positively with malignant cell abundance and negatively with multiple anti-tumor immune cells—including CD4+ T cells, NK cells, and B cells in CRC1, as well as macrophages across CRC1–3 samples.

Further evaluation using XCELL and CIBERSORT algorithms revealed significant negative correlations between AARS2 expression and common lymphoid progenitor cells (R = −0.17, P = 0.00037), M2 macrophages (R = −0.20, P = 2.4e−05), and CD4^+^ memory T cells (R = −0.21, P = 5.1e−06) (**Figure 5D**), suggesting that elevated AARS2 expression is associated with reduced infiltration of lymphoid progenitors, immunosuppressive M2 macrophages, and adaptive immune memory cells—collectively indicating a potential role for AARS2 in shaping an immunosuppressive tumor microenvironment.

We further explored the relationship of AARS2 with immunogenicity and DNA damage levels to elucidate its potential effects on immune evasion and genomic instability (**Figure 5E**). Given the relevance of immunomodulatory molecules in cancer therapy, we also analyzed their association with AARS2 to delineate an immune landscape linked to AARS2 activity (**Figure 5F**).

### 3.6 AARS2-Mediated Tumor Phenotypes and Functional Pathways in COAD

**Figure 6:**
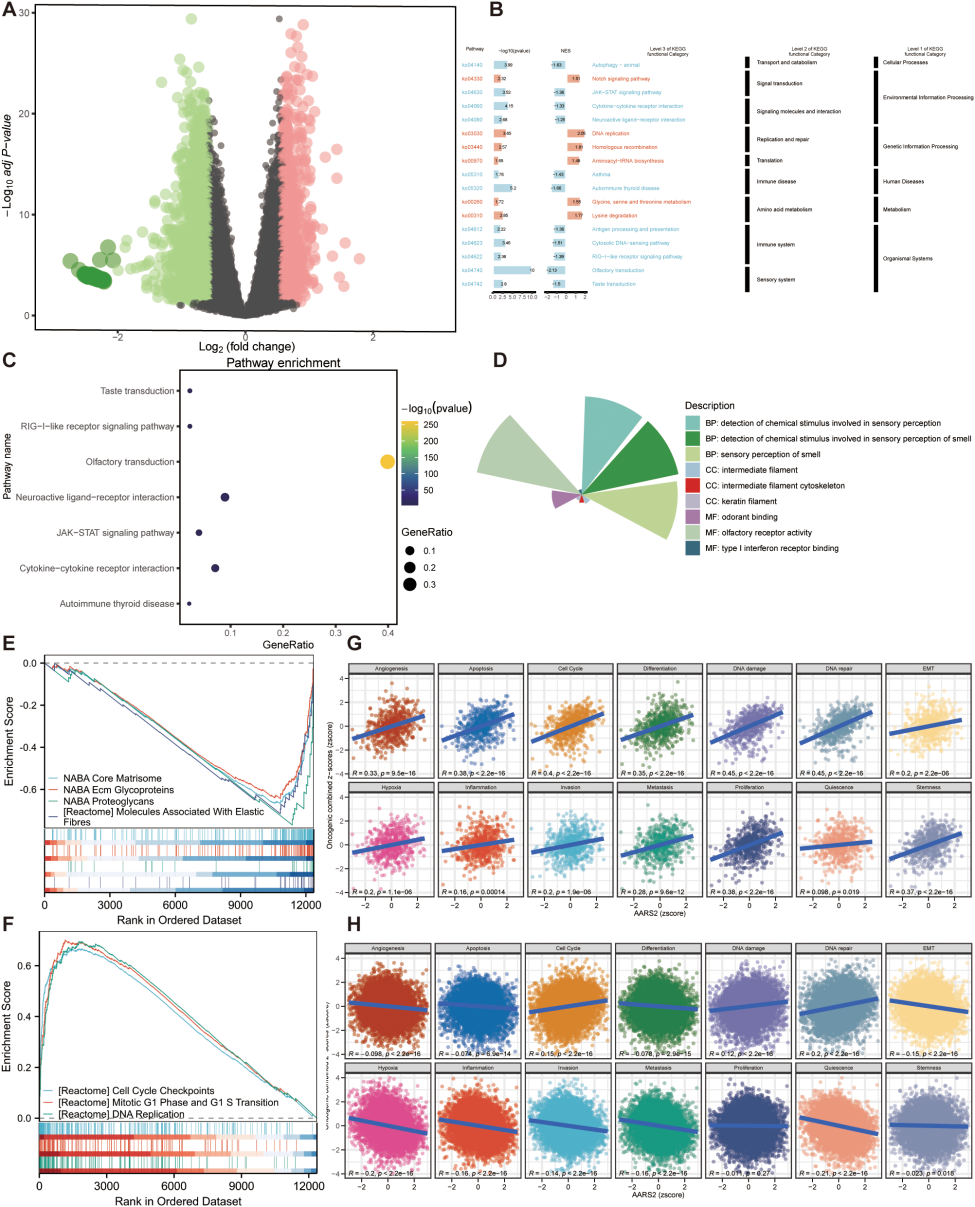
Functional analysis of AARS2 in COAD. **(A)** Differential expression analysis of COAD samples grouped by AARS2 expression, showing the identification and visualization of DEGs. **(B)** Multi-level and multi-gene set KEGG pathway profiling between AARS2 high- and low-expression groups. **(C)** KEGG enrichment analysis of DEGs in AARS2 high-expression group; **(D)** GO enrichment analysis of DEGs in AARS2 high-expression group; **(E)** GSEA Hallmark analysis showing downregulated pathways in AARS2 high-expression group; **(F)** GSEA Hallmark analysis showing upregulated pathways in AARS2 high-expression group; **(G)** Correlation between AARS2 expression and cancer-related phenotypes in COAD by GSVA; **(H)** Pan-cancer analysis of correlation between AARS2 expression and tumor-related biological processes.

To investigate the biological function of AARS2 in COAD, we first divided the COAD samples into two groups based on the median expression of AARS2 and performed differential expression analysis. This analysis identified both upregulated and downregulated DEGs. Using the thresholds of |log2FoldChange|>1 and P< 0.05, we screened the DEGs and visualized the results (**Figure 6A**). Analysis of the KEGG pathway in multi-level and multi-gene sets revealed significant differences in multiple gene sets related to COAD between the AARS2 high-expression and low-expression groups. Notably, several pathways under Genetic Information Processing were enriched, including DNA replication, homologous recombination, and aminoacyl-tRNA biosynthesis. Furthermore, visualization via a hierarchical clustering diagram intuitively demonstrated the enrichment scores and statistical significance levels of these distinct gene sets (**Figure 6B**). Subsequent KEGG enrichment analysis revealed that these genes were mainly involved in processes such as the taste transduction, RIG-I-like receptor signaling pathway, olfactory transduction, JAK-STAT signaling pathway, cytokine-cytokine receptor interaction, and autoimmune thyroid disease (**Figure 6C**). GO analysis revealed that genes highly expressed in ARRS2-positive malignant cells were primarily associated with sensory perception, including detection of chemical stimuli involved in sensory perception of smell, olfactory receptor activity, and odorant binding. Additionally, these genes were enriched in terms related to intermediate filament organization, such as intermediate filament cytoskeleton and keratin filament assembly, as well as type I interferon receptor binding (**Figure 6D**). Additionally, GSEA using Hallmark gene sets indicated that pathways related to NABA Core Matrisome, NABA ECM Glycoproteins, NABA Proteoglycans, and Molecules Associated with Elastic Fibres were downregulated in the AARS2 high-expression group (**Figure 6E**). In contrast, pathways associated with Cell Cycle Checkpoints, Mitotic G1 Phase and G1-S Transition, and DNA Replication were upregulated in this group (**Figure 6F**). GSVA was conducted to evaluate the association between AARS2 expression and COAD-related phenotypes. The results demonstrated that AARS2 expression significantly correlated with multiple malignant processes in COAD, including angiogenesis (R = 0.33, P = 9.5e−16), apoptosis (R = 0.38, P < 2.2e−16), cell cycle (R = 0.40, P < 2.2e−16), DNA repair (R = 0.35, P < 2.2e−16), epithelial-mesenchymal transition (R = 0.45, P < 2.2e−16), hypoxia (R = 0.45, P < 2.2e−16), inflammation (R = 0.20, P = 2.2e−16), invasion (R = 0.20, P = 1.1e−06), metastasis (R = 0.16, P = 0.00014), proliferation (R = 0.20, P = 1.9e−06), quiescence (R = 0.28, P = 1.9e−06), stemness (R = 0.38, P < 2.2e−16), differentiation (R = 0.38, P < 2.2e−16), and DNA damage (R = 0.37, P < 2.2e−16) (**Figure 6G**). Furthermore, a pan-cancer analysis was conducted to evaluate the correlation between AARS2 expression and multiple cancer-related phenotypes across various tumor types. The results indicated that AARS2 expression was significantly associated with various tumor-related biological processes, including angiogenesis (R = -0.098, P < 2.2e-16), apoptosis (R = -0.074, p = 6.9e-14), cell cycle (R = 0.15, P < 2.2e-16), differentiation (R = 0.12, P < 2.2e-16), DNA damage (R = 0.20, P < 2.2e-16), DNA repair (R = -0.15, p < 2.2e-16), EMT (R = -0.21, P < 2.2e-16), hypoxia (R = -0.18, P < 2.2e-16), inflammation (R = -0.10, P < 2.2e-16), invasion (R = -0.13, P < 2.2e-16), metastasis (R = -0.08, P = 1.2e-09), proliferation (R = 0.14, P < 2.2e-16), quiescence (R = 0.05, p = 0.003), and stemness (R = -0.023, P = 0.018) (**Figure 6H**).

### 3.7 The expression of AARS2 in pan-cancer tissues and PPI network

**Figure 7:**
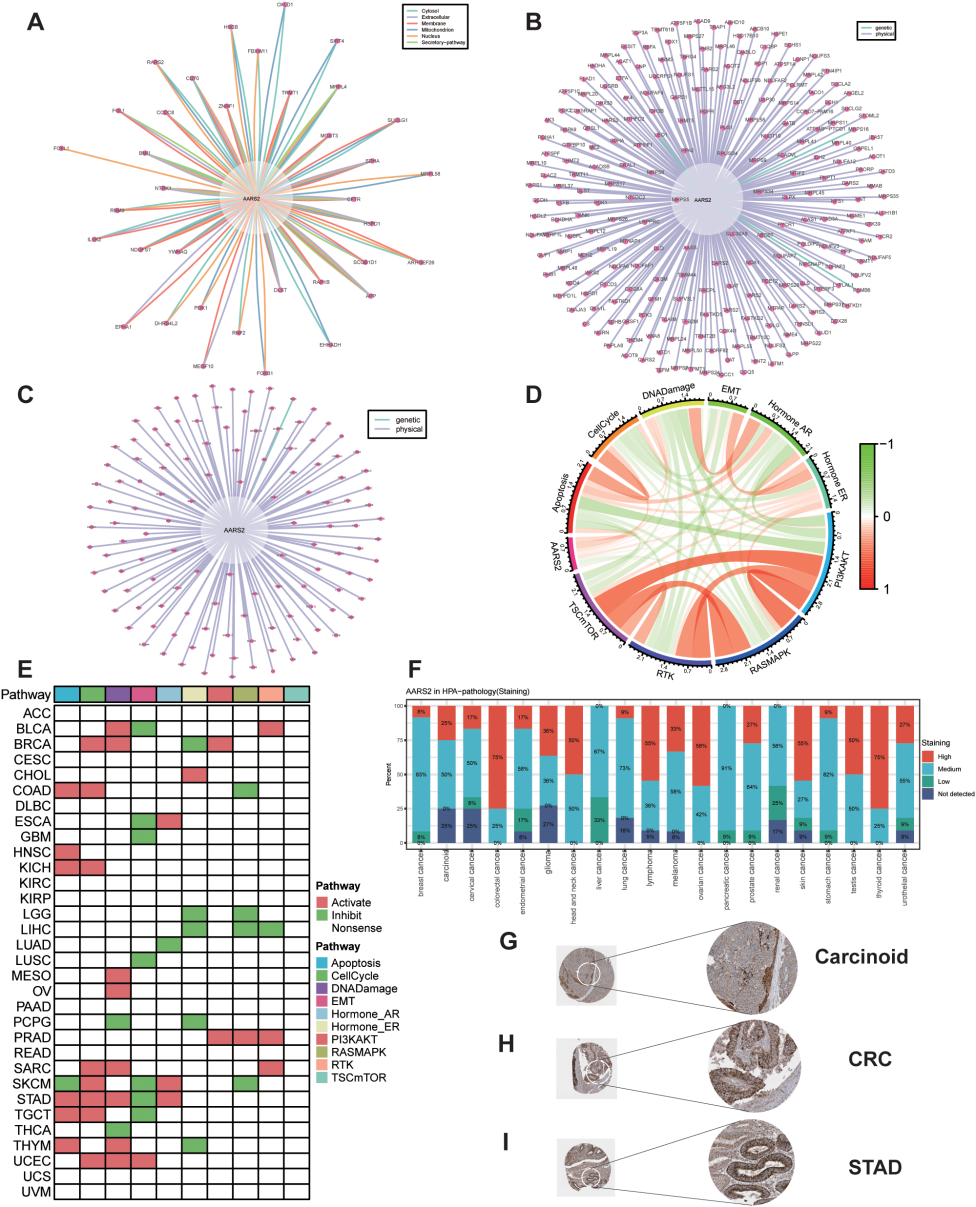
The protein expression level of AARS2 in pan-cancer and PPI network. **(A-C)** PPI network analysis of AARS2-interacting proteins using STRING and BioGRID databases. **(D)** Correlation analysis between AARS2 expression and activity of multiple cancer-related pathways in COAD. **(E)** Pan-cancer profiling of pathway activity alterations associated with high AARS2 expression across multiple cancer types. **(F)** Immunohistochemical staining patterns of AARS2 across various cancer types from HPA database. **(G-I)** Representative immunohistochemical images of AARS2 expression in carcinoid tumors, CRC and STAD.

To further investigate the interactions involving AARS2-encoded protein and to identify hub genes, we constructed a protein-protein interaction (PPI) network using the STRING database and identified proteins potentially interacting with AARS2 (**Figure 7A**). Using AARS2 as the source protein, screening and analysis via the BioGRID database revealed numerous proteins that interact with AARS2, primarily through physical interactions (**Figure 7B**). Similarly, when AARS2 was set as the target protein, multiple interacting partners were also identified (**Figure 7C**). These findings suggest that AARS2 may play a role in the assembly and functional regulation of multiple protein complexes within the cell.

Using protein expression data from the TCPA database, we performed Spearman correlation analysis between AARS2 and activity scores of 10 cancer-related pathways previously established in published studies. The results revealed a significant positive correlation between AARS2 expression and the apoptotic pathway in COAD (**Figure 7D**). Using the TCPA database and based on published research results, pathway activity scores of 10 cancer-related pathways—including TSC/mTOR, RTK, RAS-MAPK, PI3K-AKT, hormone ER, hormone AR, EMT, DNA damage response, cell cycle, and apoptosis—were calculated for pan-cancer analysis. The analysis was performed in the high AARS2 expression group across 32 tumor types: ACC, BLCA, BRCA, CESC, CHOL, COAD, DLBC, ESCA, GBM, HNSC, KICH, KIRC, KIRP, LGG, LIHC, LUAD, LUSC, MESO, OV, PAAD, PCPG, PRAD, READ, SARC, SKCM, STAD, TGCT, THCA, THYM, UCEC, UCS, and UVM. The results showed that the activities of the Apoptosis and CellCycle pathways were significantly higher in the high AARS2 expression group (**Figure 7E**).

We also obtained relevant immunohistochemical staining images from the HPA database to evaluate the expression patterns of the AARS2 gene across various tumor types. Our analysis revealed significant differences in AARS2 expression among different cancers. Specifically, colorectal cancer, thyroid cancer, lymphoma, ovarian cancer, and skin cancer exhibited a relatively high proportion of strong staining (denoted in red), accounting for 75%, 75%, 55%, 58%, and 55% of cases, respectively, indicating high AARS2 expression in these malignancies. In contrast, breast cancer, liver cancer, lung cancer, pancreatic cancer, and prostate cancer showed a predominant moderate staining pattern (denoted in blue), with proportions of 83%, 67%, 73%, and 91%, respectively, suggesting intermediate expression levels of AARS2 in these tumors (**Figure 7F**). Meanwhile, immunohistochemical data obtained from the HPA database demonstrated that AARS2 is highly expressed in carcinoid tumors, colorectal cancer (CRC), and stomach adenocarcinoma (STAD). The staining intensity and distribution patterns further revealed its predominant intracellular localization (**Figures 7G–I**).

### 3.8 Clinical samples were collected to detect the expression of AARS2 protein

**Figure 8:**
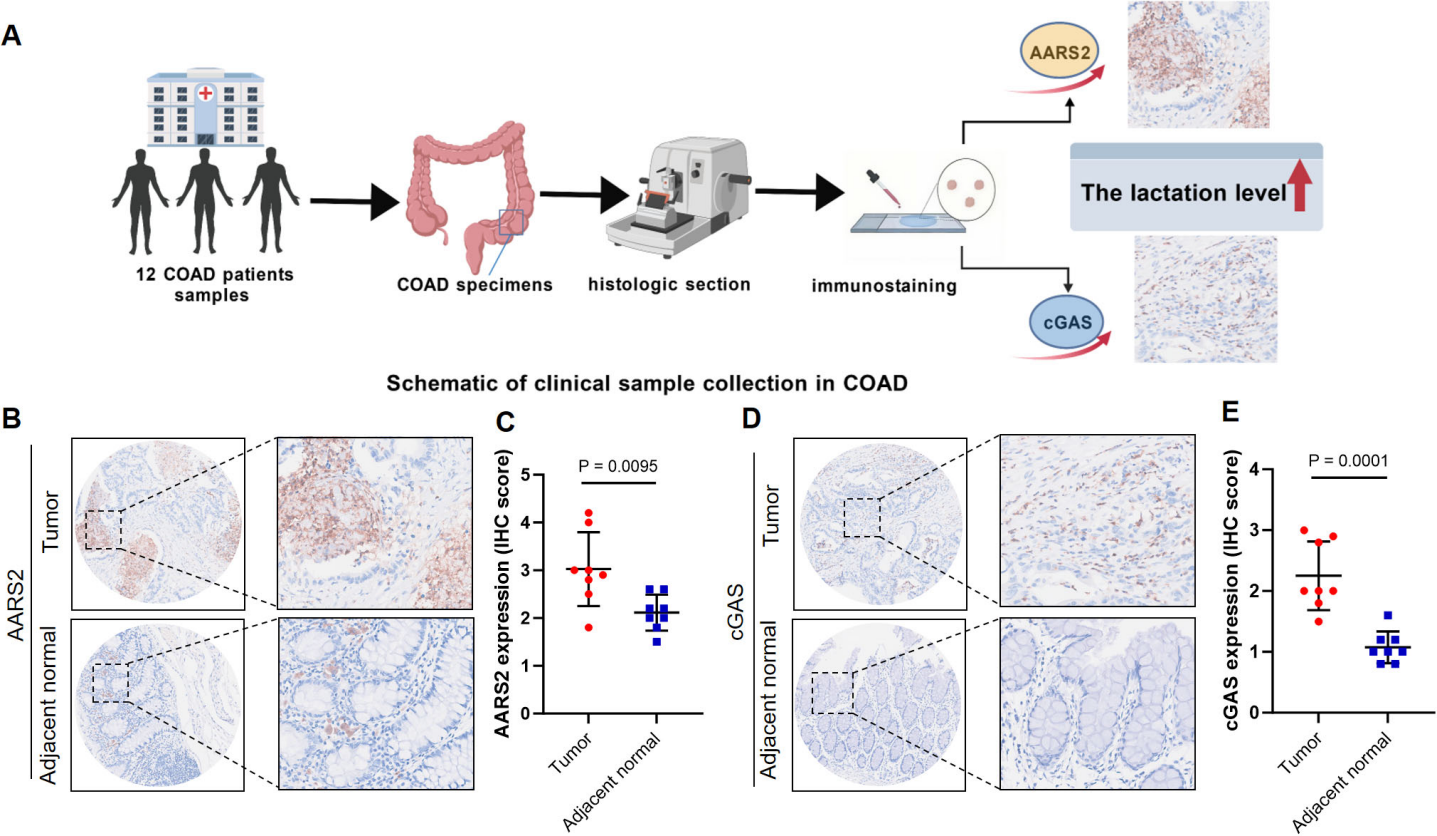
Expression of AARS2 and cGAS in human COAD tissues and control tissues. **(A)** Schematic overview of the clinical sample collection and processing workflow for the COAD cohort. **(B)** AARS2 expression is significantly higher in COAD tissues compared to adjacent tissues. **(C)** Quantitative analysis of **(B, D)** The cGAS expression is significantly higher in COAD tissues compared to adjacent tissues. **(E)** Quantitative analysis of **(D)**. **P < 0.01, ***P < 0.001.

Leveraging a clinically annotated tissue cohort from our institution—comprising surgically resected specimens from 12 COAD patients along with matched adjacent normal mucosa as internal controls (**Figure 8A**)—we first evaluated AARS2 protein expression via immunohistochemistry (IHC). IHC analysis demonstrated that AARS2 immunoreactivity was significantly stronger in COAD tissues than in adjacent normal mucosa (**Figure 8B**). Quantitative IHC scoring corroborated this observation, revealing a statistically significant increase in AARS2 expression in tumor tissues (**Figure 8C**; P < 0.01).

In parallel, immunohistochemical analysis of cGAS revealed significantly elevated protein levels in COAD tumor tissues relative to adjacent normal mucosa (**Figure 8D**), with quantitative assessment confirming this upregulation (**Figure 8E**; P < 0.001). Notably, increased cGAS protein abundance does not equate to enhanced functional activity, as cGAS signaling is primarily governed by post-translational modifications (e.g., lysine lactylation) rather than expression quantity. Consequently, the observed elevation in cGAS protein levels in clinical specimens reflects a quantitative change that requires functional validation. Subsequent *in vitro* experiments were therefore designed to specifically interrogate the activity status of the cGAS–STING pathway and its regulatory relationship with AARS2.

### 3.9 AARS2 knockdown efficiency at transcript and protein levels

**Figure 9:**
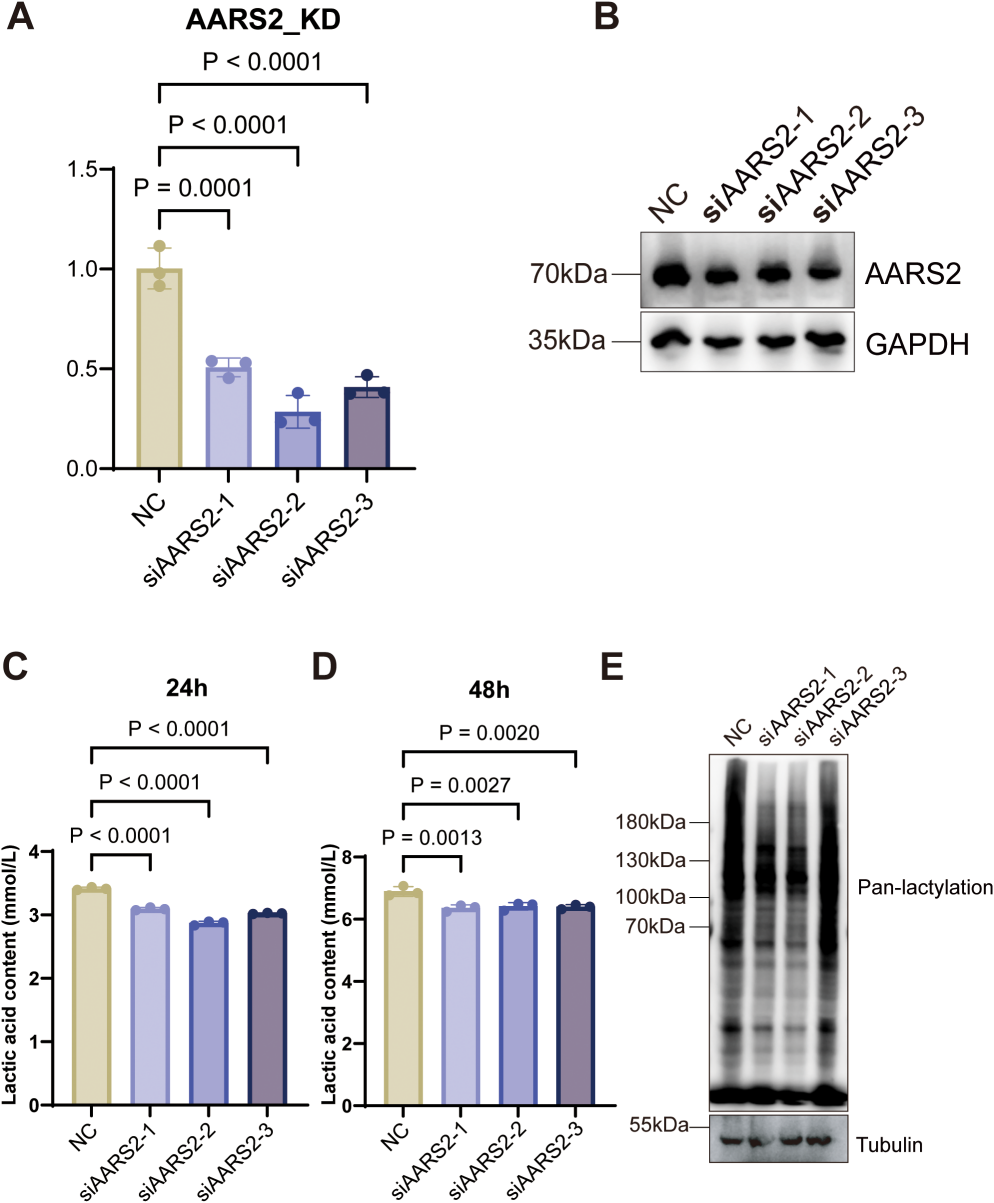
Knockdown of AARS2 suppresses lactate production and protein lactylation while upregulating downstream effectors of the cGAS pathway in HCT116 cells. **(A)** qRT-PCR validation of AARS2 knockdown efficiency using three siRNAs (siAARS2-1, siAARS2-2, siAARS2-3). **(B)** Western blot showing reduced AARS2 and elevated cGAS protein levels in knockdown groups. **(C, D)** Extracellular lactate concentrations in culture supernatants at 24 h and 48 (h) **(E)** Pan-lactylation levels; reduced in siAARS2–1 and siAARS2–2 groups, unchanged in siAARS2-3.

To elucidate the biological function of AARS2 in COAD, we established loss-of-function models in HCT 116 cells using three distinct small interfering RNAs (siAARS2-1, siAARS2-2, and siAARS2-3). The knockdown efficiency was successfully verified at both the transcriptional and translational levels. As shown in **Figures 9A**, qRT-PCR analysis demonstrated that AARS2 mRNA levels were significantly downregulated in all three knockdown groups compared to the negative control (NC) group (P<0.0001). Consistent with the mRNA data, Western blot analysis confirmed a marked reduction in AARS2 protein expression (**Figure 9B**).

Given the association between AARS2 and metabolic processes, we further investigated whether AARS2 depletion affects the glycolytic phenotype of COAD cells. Analysis of cell culture supernatants revealed that lactate production was significantly attenuated following AARS2 knockdown. Compared to the NC group, extracellular lactate concentrations were consistently reduced in the siAARS2-1, siAARS2-2, and siAARS2–3 groups at both 24 h (**Figure 9C**) and 48 h (**Figure 9D**) time points.

Since intracellular lactate levels are closely linked to protein lactylation, a newly identified post-translational modification, we examined global lactylation levels using a pan-lactylation antibody. Western blot results indicated that AARS2 silencing led to a reduction in total protein lactylation, which was most pronounced in the siAARS2–1 and siAARS2–2 groups (**Figure 9E**). Although the siAARS2–3 group exhibited a less distinct decrease in pan-lactylation signals despite reduced secreted lactate, the overall trend suggested that AARS2 is essential for maintaining high levels of intracellular lactylation.

Based on the premise that lactylation negatively regulates cGAS activity, we hypothesized that the observed reduction in lactate metabolism and protein lactylation upon AARS2 knockdown would restore cGAS signaling functionality. To validate this, we assessed the mRNA expression of key downstream effectors of the cGAS-STING pathway, including type I interferons and proinflammatory chemokines. Consistent with our hypothesis, AARS2 silencing elicited a robust activation of the innate immune response. As illustrated in **Supplementary Figure 3**, the expression levels of CCL5 (**Supplementary Figure 3A**), CXCL10 (**Supplementary Figure 3B**), and IFNB1 (**Supplementary Figure 3C**) were significantly upregulated in the siAARS2-1, siAARS2-2, and siAARS2–3 groups compared to controls (all P<0.05).

## 4 Discussion

COAD is a common malignant tumor of the digestive tract that originates in the colon. It ranks as the third most prevalent gastrointestinal malignancy globally (48, 49). Incidence rates are highest in the 40–50 year age group (50–52). COAD development is influenced by a combination of well-established genetic and environmental risk factors, including diet, adenomatous polyps, and familial adenomatous polyposis (50, 53).

Notably, the emerging epigenetic modification of lactylation may serve as a functional link between certain risk factors and cancer progression, as it plays a crucial role in regulating key biological processes such as gene expression, immune response, and tumor development (21, 54). For instance, lactylation, a novel and biologically significant PTM, has been demonstrated to serve as a critical link between metabolic reprogramming and epigenetic regulation across various pathological conditions (25, 55, 56). Research indicates that in activated hepatic stellate cells (HSCs), histone lactylation-mediated gene activation is dependent on the upregulation of hexokinase 2 (HK2) expression, thereby revealing a mechanistic pathway through which lactylation modulates both metabolic and transcriptional programs during fibrotic progression (57). In addition to histones, lactylation also modifies non-histone proteins, representing a pivotal expansion in the understanding of its regulatory breadth (58). A recent study has identified a previously unrecognized lactylation site on ALKBH5, elucidating its role in regulating m6A demethylation and enhancing antiviral innate immune responses (59). Moreover, lactylation has been implicated in the pathogenesis of multiple diseases, including sepsis, where lactate-induced lactylation of HMGB1 aggravates endothelial dysfunction (60), as well as neurodegenerative disorders and metabolic syndrome (61–63). T In oncology, elevated lactylation levels are significantly associated with poorer overall survival and disease progression in gastric cancer (GC) (64). Furthermore, lactylation has been recognized as a key driver of tumorigenesis in COAD (65), highlighting its central role in tumor metabolic reprogramming, malignant transformation, and immune evasion (66–68).

At the molecular level, cellular lactylation is a post-translational modification driven by lactic acid—a major glycolytic metabolite transported across cell membranes via monocarboxylate transporters (MCTs)—which participates in immune modulation, angiogenesis, and tissue remodeling (69–71). This modification is catalyzed by lactate dehydrogenase and histone lysine lactyltransferases such as p300/CBP, which covalently attach lactyl moieties to lysine residues on target proteins, including histones (72, 73). Emerging evidence further suggests an association between the mitochondrial gene AARS2 and lactylation processes (74). However, comprehensive investigations into AARS2 expression in COAD and its prognostic value remain limited, underscoring a gap in the current understanding of the lactylation regulatory network.

The primary role of AARS2 is to charge mitochondrial tRNA with its cognate amino acid, alanine, during translation (15, 75, 76). However, recent studies have revealed that AARS2 can also sense the intracellular lactate level and directly catalyze the lysine lactylation modification of proteins (15, 77). Once considered merely a metabolic waste product, lactate is now recognized as a key substrate for protein lactylation—a post-translational modification that enables its function as a signalling molecule and immune modulator (15, 78). The Warburg effect is a cancer hallmark defined by lactate production (79). This lactylation the tumor microenvironment, fostering immunosuppression and drug resistance through complex feedback mechanisms (80). Additionally, cGAS (cyclic guanosine monophosphate-adenosine monophosphate synthase) is a cytoplasmic DNA sensor and plays a core role in activating the innate immune response (73, 81). When cGAS recognizes DNA from colon cancer cells, it synthesizes the second messenger cGAMP, which then activates the STING signaling pathway, ultimately inducing the production of cytokines such as type I interferons (IFN), initiating anti-tumor immune responses (81–83). Some studies have shown that AARS2 can mediate the lactylation modification of cGAS (77). For example, AARS2 catalyzes the lactylation modification of a specific lysine site (such as the Lys131 of human cGAS) of cGAS, thereby directly interfering with the binding ability of cGAS to DNA (77). After lactylation, the ability of cGAS to undergo liquid-liquid phase separation after binding to DNA is impaired, and its activation ability is also impaired (81–83). The lactylation modification ultimately inhibits the enzymatic activity of the cGAS enzyme that synthesizes cGAMP, blocking the activation of the downstream STING pathway, reducing the production of type I interferons, and weakening the anti-tumor innate immune response, thereby enabling tumor cells to achieve immune evasion (78, 84).

This study employed an integrated bioinformatic and experimental approach to investigate the potential involvement of AARS2 in COAD pathogenesis. Our analyses reveal a significant association between elevated AARS2 expression and adverse clinical features specifically in COAD cohorts, suggesting its potential value as a prognostic indicator within this malignancy. Multi-omics profiling further indicates that AARS2 may participate in COAD-relevant biological processes—including metabolic adaptation, immune modulation, and tumor microenvironment remodeling—extending beyond its canonical role in mitochondrial translation. We acknowledge that the initial univariate Cox analysis in COAD lacked multiple testing correction—a deliberate choice to preserve a biologically inclusive candidate pool for rigorous downstream validation, as stringent correction would have excluded potentially relevant signals. Crucially, AARS2 emerged as the top consensus candidate across multiple algorithms and was robustly validated through independent cohorts, multi-omics profiling, immunohistochemistry, and functional assays, substantially mitigating false-positive concerns for this specific gene. While other screened candidates require further investigation, the convergent evidence supporting AARS2 remains scientifically sound.

Transcriptomic analyses of COAD datasets identified a negative correlation between AARS2 expression and interferon-related signaling signatures. To functionally explore this association in the COAD context, we performed loss-of-function experiments in the COAD-derived cell line HCT116. AARS2 knockdown significantly reduced extracellular lactate production and global protein lactylation while concurrently upregulating key cGAS–STING downstream effectors (CCL5, CXCL10, IFNB1). These findings support a plausible model wherein AARS2, within the COAD metabolic landscape, influences lactate availability and the lactylation milieu, potentially contributing to attenuated innate immune signaling in tumor cells. Immunohistochemical validation in 12 paired COAD clinical specimens confirmed elevated protein expression of both AARS2 and cGAS in tumor tissues versus adjacent mucosa. Critically, given that cGAS activity in COAD is governed primarily by post-translational modifications rather than protein abundance, the observed cGAS upregulation in tumor tissues may reflect a compensatory mechanism; however, due to the limited sample size (n=12), this finding could equally represent a stochastic observation. The concurrent restoration of interferon signaling upon AARS2 depletion in COAD-derived cells provides preliminary, context-dependent support for the compensatory hypothesis, yet definitive interpretation requires validation in larger, well-annotated COAD cohorts and dedicated mechanistic studies to clarify the biological relevance of this expression pattern within colon cancer pathogenesis.

We explicitly clarify that our COAD-focused data do not establish AARS2 as a direct lactyltransferase or as the enzyme catalyzing site-specific cGAS lactylation (e.g., K131) in colon cancer. Current evidence positions AARS2 primarily as a mitochondrial alanyl-tRNA synthetase; its association with lactylation in COAD appears indirect, likely mediated through metabolic regulation of lactate pools. Consistent with prior studies (77), we propose that in the COAD microenvironment, AARS2 may foster an immunosuppressive niche by sustaining lactate-Associated lactylation dynamics that potentially dampen cGAS–STING activity. GSVA of COAD transcriptomes further linked high AARS2 expression to hypoxia and epithelial-mesenchymal transition signatures—processes well-documented to promote immunosuppression in colorectal carcinogenesis. While biologically coherent, these associations remain correlative and require deeper mechanistic validation within COAD-specific models.

We acknowledge several important limitations. First, although AARS2 knockdown reduced global lactylation and restored interferon-related gene expression, we did not directly assess site-specific lactylation of cGAS (e.g., via K131-lactylation immunoblotting, mass spectrometry, or co-immunoprecipitation). Thus, whether AARS2 influences cGAS activity via direct lactylation of cGAS—or through broader metabolic or indirect regulatory mechanisms—remains unresolved. Second, functional validation was confined to a single COAD cell line (HCT116); validation across diverse cellular contexts and *in vivo* models is essential. Third, the clinical cohort (n=12 paired samples) is limited in size and lacks longitudinal treatment/outcome data, precluding robust survival analysis. Fourth, while bioinformatic analyses implicated AARS2 in immune evasion, the precise cellular sources (tumor vs. stromal) and immunological consequences within the tumor microenvironment warrant spatial and single-cell resolution studies. Finally, the molecular basis linking AARS2 to lactate metabolism—whether through transcriptional regulation of glycolytic enzymes, mitochondrial function, or other pathways—requires further investigation. Future work should prioritize the direct detection of cGAS lactylation following AARS2 modulation, complemented by rescue experiments involving lactate supplementation or lactylation-mimetic cGAS mutants. Validation efforts should be expanded to patient-derived organoids and immunocompetent animal models, while concurrently investigating AARS2’s potential non-canonical functions beyond its established role in mitochondrial tRNA synthesis. Furthermore, further experimental validation at the single-cell level, specifically dissecting the precise functional impact of AARS2 on different T helper cell subsets (Th1, Th2, Th17, Treg) within the COAD TME, would provide deeper mechanistic insights into its role in immune modulation. These integrated approaches are essential to clarify mechanistic relationships and assess translational potential.

In summary, this study positions AARS2 as a metabolism-linked candidate gene associated with lactylation dynamics and suppressed innate immunity in COAD. While causal claims regarding AARS2- associated lactylation or direct cGAS modification are not substantiated by current data, our integrated findings provide a testable framework for future mechanistic inquiry. These insights may inform the development of metabolism-targeted strategies to reverse immunosuppression in colorectal cancer, emphasizing the need for rigorous validation before translational application.

## 5 Conclusion

In conclusion, our study identifies AARS2 as a candidate gene associated with lactate metabolism dysregulation in colon adenocarcinoma. Integrated bioinformatic and preliminary experimental evidence suggests a potential link between AARS2 expression, global protein lactylation, and cGAS–STING pathway suppression. While these findings highlight AARS2 as a hypothesis-generating biomarker worthy of further investigation, we emphasize that current data remain correlative and lack direct mechanistic validation (e.g., site-specific cGAS lactylation). The observed associations require rigorous confirmation in larger cohorts, diverse models, and functional assays. This work provides a cautious yet actionable foundation for exploring metabolism-immune crosstalk in COAD, underscoring the necessity of methodological rigor in translating associative insights toward clinical relevance.

## Data Availability

The datasets presented in this study can be found in online repositories. The names of the repository/repositories and accession number(s) can be found in the article/**Supplementary Material**.
