# Supplementary figures and images for: Integrative pan-cancer analysis reveals AARS2 as a lactylation-associated biomarker and therapeutic target in colon adenocarcinoma
Source: Front Immunol. 2026 Feb 27;17:1732811. doi: 10.3389/fimmu.2026.1732811 (PMC12982081; doi:10.3389/fimmu.2026.1732811)

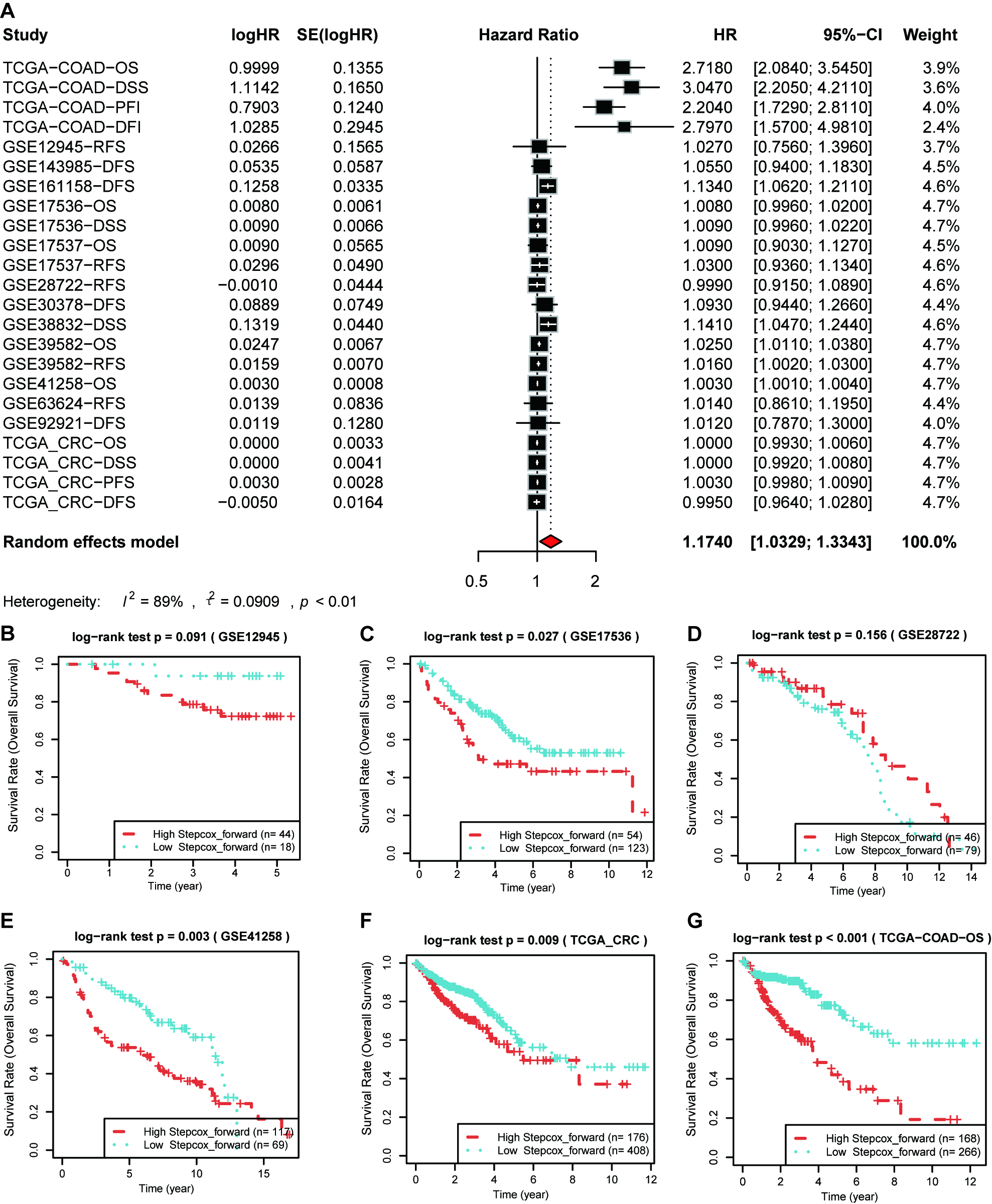

Supplement: Supplementary file 1 [file Image1.tif]

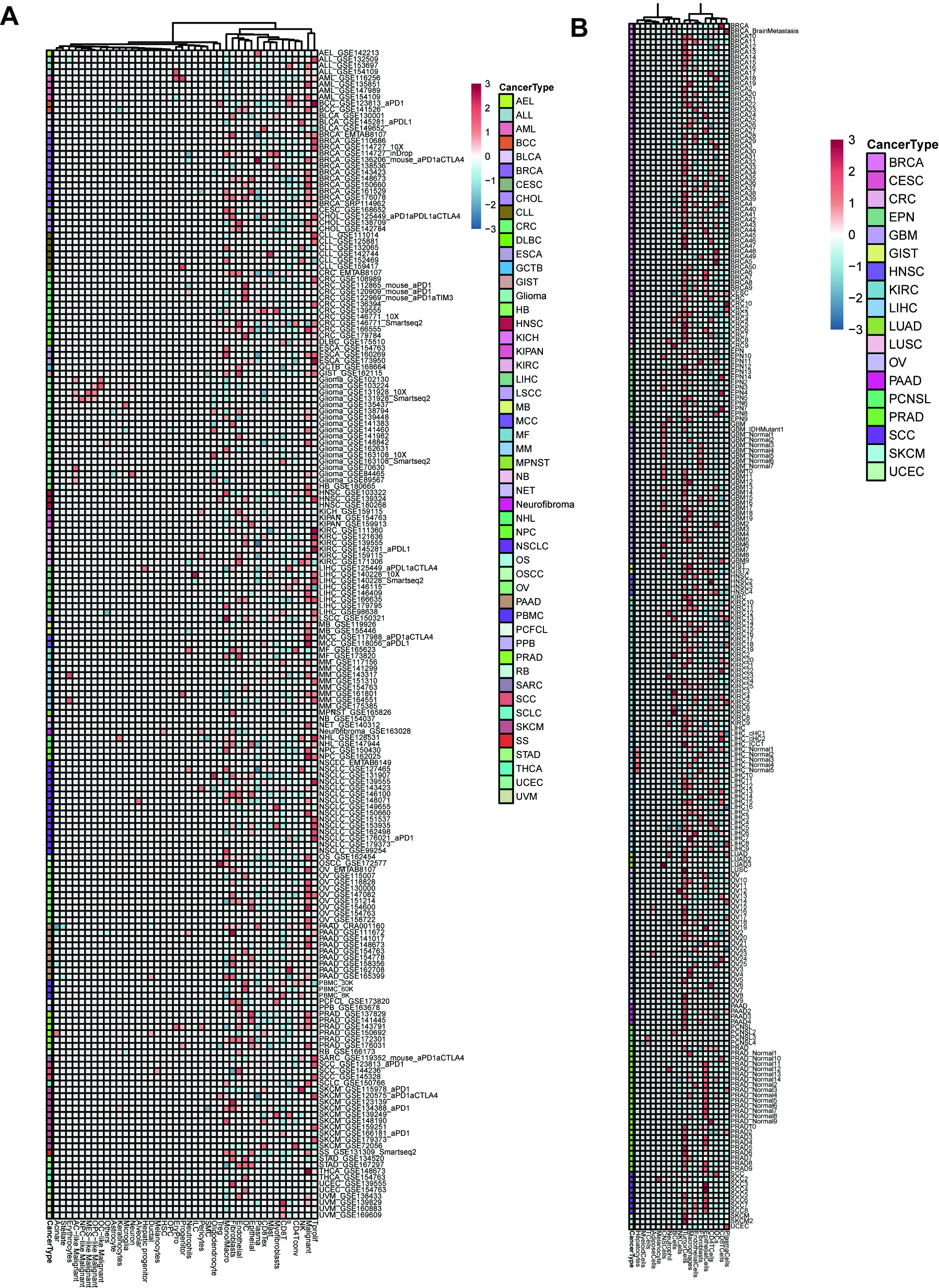

Supplement: Supplementary file 2 [file Image2.tif]

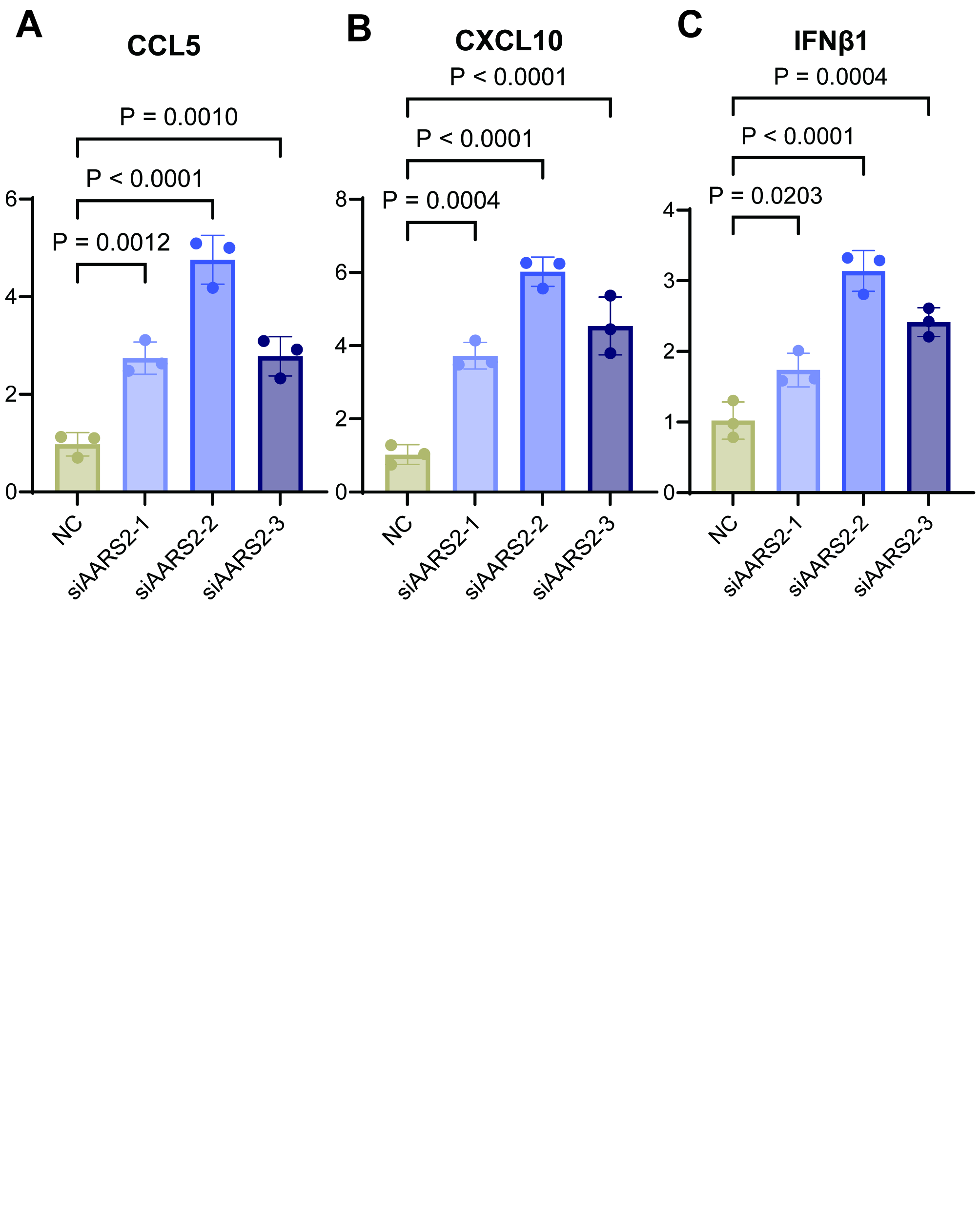

Supplement: Supplementary file 3 [file Image3.tif]
