# Supplementary material for: Integrative pan-cancer analysis reveals AARS2 as a lactylation-associated biomarker and therapeutic target in colon adenocarcinoma
Source: Front Immunol. 2026 Feb 27;17:1732811. doi: 10.3389/fimmu.2026.1732811 (PMC12982081; doi:10.3389/fimmu.2026.1732811)
Supplement: Supplementary file 5 [file Table2.docx]

**Supplementary Table S1. Clinicopathological characteristics of IHC patients.**

| **No.** | **Sex** | **Age** | **Tumor Location** | **Stage** | **T** | **N** | **M** | **MSI Status** |
| --- | --- | --- | --- | --- | --- | --- | --- | --- |
| 1 | F | 58 | Hepatic flexure colon | III | 3 | 1 | 0 | MSS |
| 2 | M | 72 | Rectum | II | 4 | 0 | 0 | MSS |
| 3 | F | 67 | Rectum | II | 3 | 0 | 0 | MSS |
| 4 | M | 67 | Rectum | III | 4 | 1 | 0 | MSS |
| 5 | M | 56 | Rectum | IV | 3 | 0 | 1 | MSS |
| 6 | M | 69 | Rectum | III | 3 | 2 | 0 | MSS |
| 7 | M | 67 | Right-sided colon | II | 3 | 0 | 0 | MSS |
| 8 | F | 74 | Right-sided colon | II | 3 | 0 | 0 | MSS |
| 9 | M | 63 | Rectum | III | 3 | 1 | 0 | MSS |
| 10 | F | 70 | Right-sided colon | II | 3 | 0 | 0 | MSS |
| 11 | M | 59 | Splenic flexure colon | II | 3 | 0 | 0 | MSS |
| 12 | F | 65 | Left-sided colon | III | 3 | 2 | 0 | MSS |
