# Supplementary material for: Integrative pan-cancer analysis reveals AARS2 as a lactylation-associated biomarker and therapeutic target in colon adenocarcinoma
Source: Front Immunol. 2026 Feb 27;17:1732811. doi: 10.3389/fimmu.2026.1732811 (PMC12982081; doi:10.3389/fimmu.2026.1732811)
Supplement: Supplementary file 6 [file Table3.docx]

**Supplementary Table S2. qPCR primer sequences**

| **Primer Name** | **Primer Sequence (5' to 3')** |
| --- | --- |
| AARS2-F | GTTCAAGCCAATCTTTCTGGGC |
| AARS2-R | CAGGTCGTTATGGTGTCCTCC |
| IFNB1-F | ATGACCAACAAGTGTCTCCTCC |
| IFNB1-R | GGAATCCAAGCAAGTTGTAGCTC |
| CXCL10-F | GTGGCATTCAAGGAGTACCTC |
| CXCL10-R | TGATGGCCTTCGATTCTGGATT |
| CCL5-F | CCAGCAGTCGTCTTTGTCAC |
| CCL5-R | CTCTGGGTTGGCACACACTT |
| GAPDH-F | GGAGCGAGATCCCTCCAAAAT |
| GAPDH-R | GGCTGTTGTCATACTTCTCATGG |
