# Supplementary material for: Integrative pan-cancer analysis reveals AARS2 as a lactylation-associated biomarker and therapeutic target in colon adenocarcinoma
Source: Front Immunol. 2026 Feb 27;17:1732811. doi: 10.3389/fimmu.2026.1732811 (PMC12982081; doi:10.3389/fimmu.2026.1732811)
Supplement: Supplementary file 7 [file Table4.docx]

**Supplementary Table S3. Univariate Cox analysis of lactylation-related genes.**

| **ID** | **HR** | **HR.95L** | **HR.95H** | **pvalue** |
| --- | --- | --- | --- | --- |
| AARS2 | 1.57689 | 1.085433 | 2.290866 | 0.01684 |
| HDAC3 | 0.42828 | 0.227171 | 0.807425 | 0.008763 |
| H4C14 | 1.460108 | 1.184578 | 1.799726 | 0.000389 |
| H4C16 | 1.315201 | 1.056047 | 1.637951 | 0.014405 |
| H4C5 | 1.279436 | 1.05557 | 1.550781 | 0.012037 |
| H2AZ1 | 0.621885 | 0.434799 | 0.88947 | 0.009283 |
| H2BC5 | 1.485408 | 1.206406 | 1.828934 | 0.000193 |
| H3C13 | 1.309104 | 1.053144 | 1.627274 | 0.015248 |
| H2BC11 | 1.328313 | 1.093712 | 1.613235 | 0.004191 |
| H2BC15 | 1.233469 | 1.008422 | 1.508739 | 0.041195 |
| H3C4 | 1.269594 | 1.059327 | 1.521597 | 0.009771 |
| H3C6 | 1.33772 | 1.077009 | 1.661542 | 0.008521 |
| H2BC4 | 1.263224 | 1.051405 | 1.517716 | 0.012586 |
| H3C10 | 1.28019 | 1.051822 | 1.558142 | 0.013742 |
| H2BC17 | 1.304184 | 1.039569 | 1.636155 | 0.021712 |
| H2BC6 | 1.338692 | 1.07898 | 1.660917 | 0.008031 |
| H2BC26 | 1.390942 | 1.118919 | 1.729098 | 0.002959 |
| H2BC7 | 1.256995 | 1.042915 | 1.51502 | 0.016347 |
| H2BC8 | 1.263347 | 1.063422 | 1.500858 | 0.007824 |
| MECP2 | 1.784746 | 1.176762 | 2.706851 | 0.006413 |
| MPC1 | 0.630086 | 0.456236 | 0.870182 | 0.005045 |
| NMNAT1 | 0.673979 | 0.467178 | 0.972321 | 0.034852 |
| ULK1 | 1.871227 | 1.261826 | 2.774939 | 0.001829 |
| TRAP1 | 0.670637 | 0.464253 | 0.968768 | 0.03325 |
| H2AC6 | 1.437298 | 1.161233 | 1.778992 | 0.000857 |
| H2AC18 | 1.312807 | 1.089974 | 1.581196 | 0.004134 |
| H2AC8 | 1.298818 | 1.088207 | 1.550189 | 0.003775 |
| H2AC7 | 1.218259 | 1.010883 | 1.468176 | 0.038111 |
| PAX6 | 1.248178 | 1.014236 | 1.53608 | 0.036309 |
| ALDOB | 0.911878 | 0.840678 | 0.989108 | 0.026149 |
| CRYAB | 1.181075 | 1.029947 | 1.354379 | 0.017202 |
| SLC4A4 | 0.905554 | 0.838448 | 0.97803 | 0.011554 |
